# Integrative multi-omic analysis reveals epigenetic and neurotransmitter signaling disruption induced by tributyltin in zebrafish eleutheroembryos

**DOI:** 10.1093/eep/dvag031

**Published:** 2026-09-24

**Authors:** Albert Menéndez-Pedriza, Janan Gawra, Jorke H Kamstra, Melissa Faria, Marina Bellot, Cristian Gómez-Canela, Demetrio Raldúa, Juliette Legler, Joaquim Jaumot, Benjamin Piña, Laia Navarro-Martín

**Affiliations:** Department of Environmental Chemistry, IDAEA-CSIC, Jordi Girona 18–26, 08034 Barcelona, Spain; PhD Program Analytical Chemistry and the Environment, University of Barcelona, 08034, Barcelona, Spain; Department of Environmental Chemistry, IDAEA-CSIC, Jordi Girona 18–26, 08034 Barcelona, Spain; Institute for Risk Assessment Sciences, Department of Population Health Sciences, Faculty of Veterinary Medicine, Utrecht University, 3584, CM Utrecht, the Netherlands; Department of Environmental Chemistry, IDAEA-CSIC, Jordi Girona 18–26, 08034 Barcelona, Spain; Department of Analytical and Applied Chemistry, School of Engineering, Institut Químic de Sarrià-Universitat Ramon Llull, Via Augusta 390, 08017, Barcelona, Spain; Department of Analytical and Applied Chemistry, School of Engineering, Institut Químic de Sarrià-Universitat Ramon Llull, Via Augusta 390, 08017, Barcelona, Spain; Department of Environmental Chemistry, IDAEA-CSIC, Jordi Girona 18–26, 08034 Barcelona, Spain; Institute for Risk Assessment Sciences, Department of Population Health Sciences, Faculty of Veterinary Medicine, Utrecht University, 3584, CM Utrecht, the Netherlands; Utrecht Institute for Pharmaceutical Sciences, Faculty of Science, Utrecht University, 3584 CG, Utrecht, The Netherlands; Department of Environmental Chemistry, IDAEA-CSIC, Jordi Girona 18–26, 08034 Barcelona, Spain; Department of Environmental Chemistry, IDAEA-CSIC, Jordi Girona 18–26, 08034 Barcelona, Spain; Department of Environmental Chemistry, IDAEA-CSIC, Jordi Girona 18–26, 08034 Barcelona, Spain

**Keywords:** environmental epigenetics, endocrine disruption, tributyltin, zebrafish embryos, multi-omics, neurotoxicity, neurotransmitters, behaviour

## Abstract

Environmental exposure to endocrine disruptors such as Tributyltin (TBT) has been established as a serious threat causing neurological impairment, reproduction dysfunction and lipid dysregulation. However, the molecular mechanisms underlying TBT-induced toxicity, particularly its capacity to disrupt epigenetic programming during critical developmental windows, remain poorly understood. State-of-the-art multi-omic approaches have emerged to consider the complexity of the different omic systems, crucial for a better characterization of the mechanisms of action of pollutants. In the present study, a novel multi-omic analysis integrating epigenomics, transcriptomics, and metabolomics was conducted on zebrafish eleutheroembryos exposed from 2 to 5 days post-fertilization to two non-toxic TBT concentrations (3 and 30 nM). To capture not only the toxic mechanisms but also multifaceted molecular interactions from gene to phenotype, multi-omics results were complemented by behavioural and neurotransmitter content analyses at the end of the exposures and after a 3-day clearance period. TBT induced the appearance of 5 280 differentially methylated regions, with significant enrichment in intergenic, CpG islands, and promoter regions. Our results also revealed that altered differentially methylated regions were involved in key biological processes, including nervous system development, synaptic signaling, and muscle structure development. Multi-omic analysis identified significant alterations in neurotransmitter signaling pathways, which were then confirmed through neurotransmitter content analysis. TBT also affected responses at the organismal level, particularly impairing cognitive performance. Altogether, the integration of multi-omic strategies demonstrated the potential to revolutionize the future of toxicology by enhancing the understanding of environmental pollutant effects, paving the way for more refined risk assessment and regulatory frameworks.

## Graphical Abstract

**Graphical Abstract:**
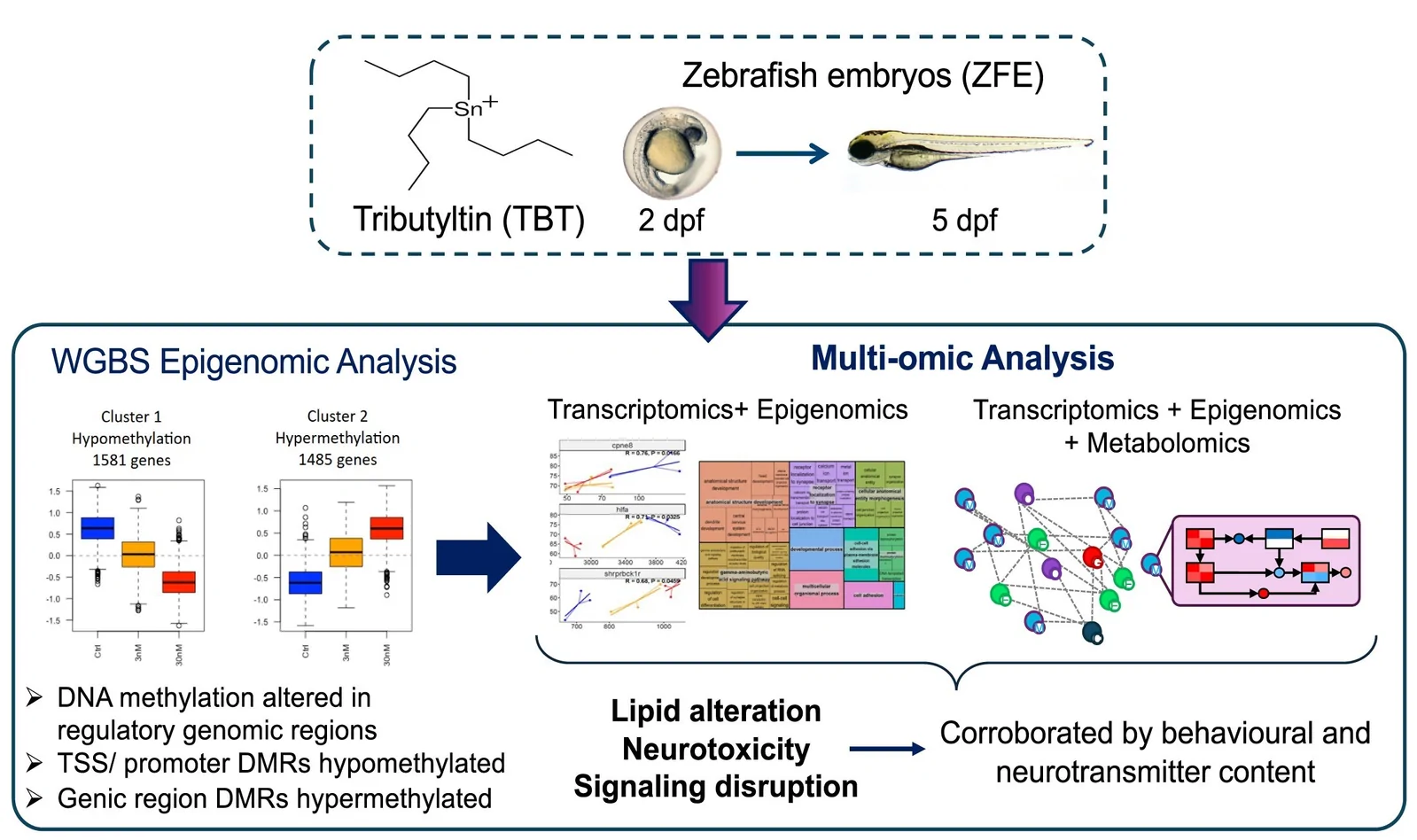

## Introduction

Endocrine-disrupting chemicals (EDCs) are exogenous compounds that can alter and modulate endocrine system homeostasis. They can disrupt the synthesis, release, and action of natural hormones, altering the physiological function of specific endogenous hormones. A wide range of potential EDCs are continuously released into the environment [1], being of particular concern organotin compounds, especially tributyltin (TBT). TBT are organometallic (Sn(C_4_H_9_)^3+^ X^−^) compounds introduced in marine ecosystems in the early 1960s for being used worldwide in industrial and agricultural biocide applications, primarily as an antifouling agent [2]. It rapidly attracted the attention of the environmental community due to its demonstrated perturbations in the survival, growth, development and reproduction of many species [3]. Although its use was banned by the International Marine Organization (IMO) in 2008, its persistence in sediments, bioaccumulation potential, and ongoing illegal release into aquatic ecosystems have allowed TBT to remain a major hazard in many coastal regions [4]. TBT exposure has been established as a serious threat due to its ability to impair reproductive function in several aquatic species leading to imposex or masculinization [5, 6]. More recent studies have highlighted additional toxicological effects of organotins in aquatic species such as fish, crustaceans and amphibians, including lipotoxicity, cardiovascular toxicity, developmental delay and endocrine-disruptive activities such as thyroid dysfunction [7–9]. Interestingly, a recent study underlined the potential influence of TBT on amino acid metabolism [10], which may result in a significant alteration of neurotransmitter content. For instance, L-tyrosine and uridine, involved in the catecholaminergic pathway, leading to the synthesis of dopamine, norepinephrine, and epinephrine, were found affected by TBT exposure. Similarly, other key metabolite related to neurotransmitters systems, such as L-glutamate, L-glutamine, L-proline and taurine, were also reported to be altered. Therefore, previous results may align with reported neurotoxicity of this organotin compound, which potentially induces behavioural alterations in aquatic species and mammals [11].

The ability of environmental pollutants to influence epigenetic programming has only recently gained attention in the scientific community. Environmental epigenetics is steadily emerging as a key field to investigate the toxicity of environmental stressors. In particular, DNA methylation has gained relevance due to its critical role in early development [12]. There is mounting evidence that environmental pollutants including persistent dioxins or polycyclic aromatic hydrocarbons (PAHs) can induce significant epigenetic alterations in ecotoxicologically relevant species [13]. Metal-induced oxidative stress has also been related to epigenetic alterations, linking the perturbation of these regions to developmental and neurological disorders [14]. Epigenetic perturbations of several EDCs have also been closely related to neurological alteration [15]. In particular, the role of TBT in disrupting epigenetic programming is still poorly characterized. However, some recent studies have demonstrated the potential of TBT to impact DNA methylation profile on several organisms such as mammals, marine gastropods and fish species. These works emphasized the growing evidence linking early-life exposure to environmental pollutants to later disruptions in neurotransmitter levels and neurobehavioural outcomes, as well as the potential of TBT to induce intergenerational neurodevelopmental toxicity [16–18]. Interestingly, Martínez *et al.*, highlighted the alteration of several biological pathways related to epigenetic programming, including methylation, histone methylation, methyltransferase activity and S-adenosyl-L-methionine (SAM), which plays a vital role in methylation reactions for being the primary methyl donor in eukaryotes, whereas Ortiz-Villanueva *et al.*, also reported the significant disruption of L-methionine, precursor of SAM (Ortiz-Villanueva et al., 2018). Other studies related the obesogenic effects of TBT and other EDCs with their interference with the epigenetic programming of gene regulation, partly through the activation of nuclear receptors involved in metabolic homeostasis [19]. Despite the increasing availability of information, the mechanisms by which EDCs such as TBT interfere with epigenetic endpoints, particularly in those pathways related to neurodevelopment and behaviour, remain unclear [20].

As in many omic disciplines, high-throughput epigenomics is shifting from single- to novel multi-omics approaches. Here, our aim was to establish a mechanistic understanding of behavioural effects induced by TBT in zebrafish embryos, using a multi-omics approach. By using such approach, we were able to integrate multiple biological layers, facilitating a more comprehensive understanding of toxicity mechanisms and key events for adverse outcome pathway (AOP) development (i.e. model for identifying the sequence of molecular and cellular events required to produce a certain toxic effect after an organism was exposed to a exogenous substance). However, despite its growing adoption in other scientific fields, particularly biomedical disciplines, the integration of environmental epigenomics with other omic approaches remains limited. In particular, the integration of gene-based (e.g. epigenomics, transcriptomics) and non-gene-based technologies (i.e. metabolomics) still presents significant challenges in multi-omic workflows, primarily within the subsequent data analysis framework. These challenges include heterogeneity across omic technologies, the treatment of missing values, and the complexity of interpreting multilayered systems models [21, 22]. Such limitations highlight the need for studies that systematically integrate epigenetic perturbations with functional phenotyping to better understand the dose-dependent effects of pollutants such as TBT. Zebrafish (*Danio rerio*), a well-established toxicological model organism, has been recognized as an excellent biosystem for studying epigenetic modifications in response to environmental stressors [23, 24]. This makes zebrafish an ideal model for multi-omic investigations of developmental neurotoxicity, where epigenetic modifications can be directly linked to functional outcomes such as behavioural deficits and neurotransmitter dysregulation.

In the present work, epigenetic alterations induced by TBT were assessed in zebrafish eleutheroembryos from 2 to 5 days post-fertilization (dpf) using an untargeted epigenomic approach (Whole Genome Bisulfite Sequencing, WGBS). Additionally, to further elucidate the biological processes disrupted by TBT, state-of-the-art multi-omic approaches were applied by integrating epigenomic data with previously obtained transcriptomic and metabolomic datasets published by our group. Also, phenotypic endpoints, including behavioural and neurotransmitter content analyses were conducted, including a clearance period to determine potential persistent effects. To the best of the authors' knowledge, although TBT is recognized as the most toxic organotin compound and a major environmental pollutant, its epigenomic effects have not yet been investigated within a multi-omic framework in any relevant animal model. This study addresses that gap by providing novel insights into the toxicological mechanisms of TBT through the integration of epigenetic, multi-omic, and phenotypic data, thereby linking molecular disruptions to organism-level outcomes. By integrating multiple molecular layers with functional phenotyping, this multi–omic framework aims not only to identify affected pathways, but also to prioritize mechanistically coherent signals that persist across biological scales, thereby increasing robustness and biological relevance compared to single–omic approaches.

## Results

A schematic overview of the experimental workflow conducted in this study is depicted in Fig. 1. As seen, for all assays conducted two different environmentally relevant TBT concentrations (3 nM and 30 nM) were evaluated. The present study applied whole–genome bisulfite sequencing (WGBS) to zebrafish embryos exposed to tributyltin (TBT) during organogenesis and integrated these newly generated methylation data into a multi-omics framework considering previously published transcriptomic and metabolomic datasets derived from the same initial exposure experiment. In addition, newly generated behavioural and neurotransmitter data obtained from independent follow–up exposures were included. Specifically, WGBS was performed on DNA preserved from the original exposure experiment in which RNA–seq and metabolomic analyses were previously reported (Batch 1), while behavioural and neurotransmitter analyses were generated in the present study using independent exposure experiments (Batch 2). For more details see Materials and Methods section.

**Figure 1:**
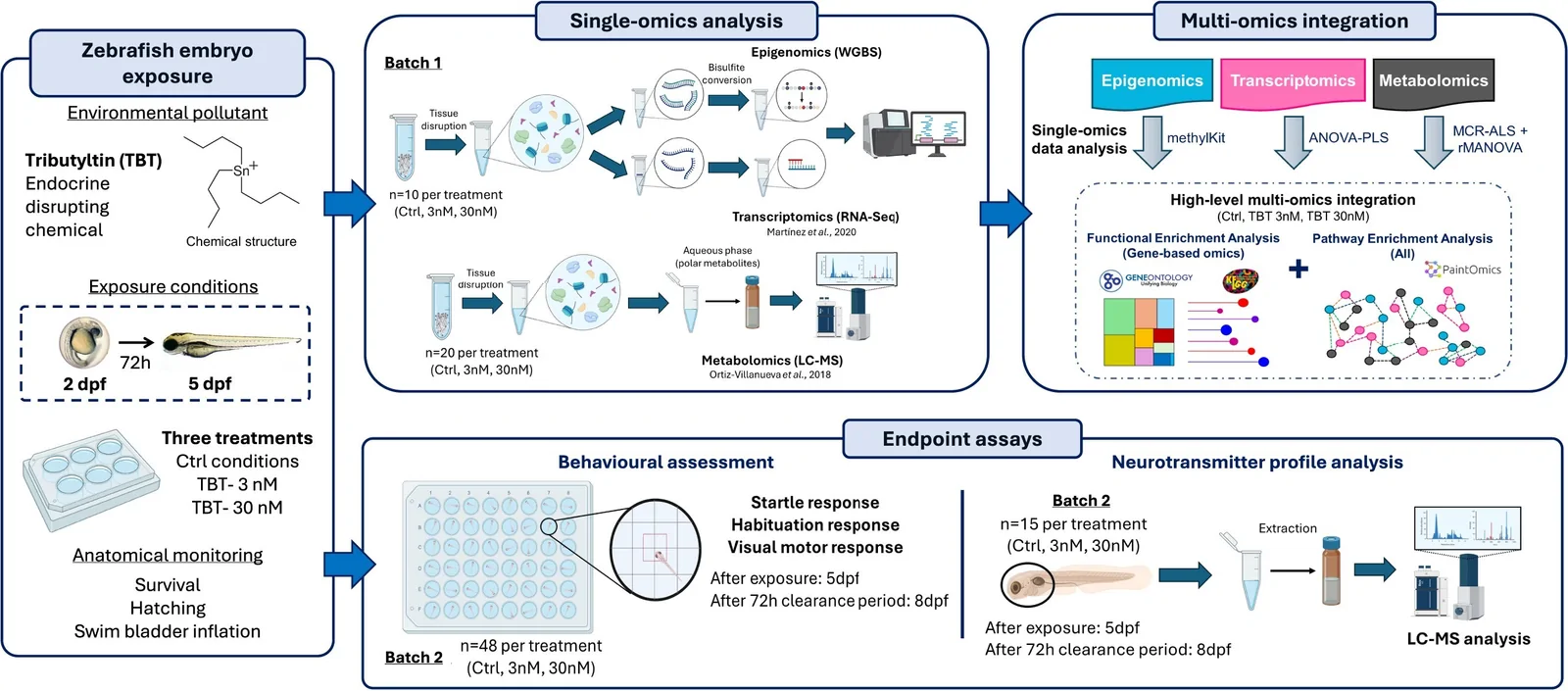
Schematic overview of the experimental workflow conducted in this study. Zebrafish embryos were exposed to tributyltin (TBT) at 3 nM or 30 nM from 2 to 5 days post-fertilization. Batch 1 samples were used for whole-genome bisulfite sequencing and integrated with previously generated transcriptomic and metabolomic datasets in a multi-omics analysis pipeline. Batch 2 samples were used for behavioural testing and neurotransmitter quantification at 5 days post-fertilization and again after a 72-hour recovery period in clean water (8 days post-fertilization). The workflow links epigenomic, transcriptomic, metabolomic, behavioural, and neurotransmitter endpoints to assess the effects of TBT exposure). Selected icons and graphical elements were created using BioRender.com

### Genome-wide DNA methylation changes induced by TBT exposure

#### Identification and genomic distribution of differentially methylated regions

DNA methylation was assessed in 5 biological replicates (pools of 10 eleutheroembryos) per treatment. A total of 22 215 DMRs were identified by comparing methylation levels across all treatment groups (Ctrl vs 3 nM, Ctrl vs 30 nM, and 3 nM vs 30 nM) using logistic regression with a sliding linear model (SLIM) as described in the methods section (Q-value thresholds of 0.01, Supplementary Table 1B). The identified DMRs were used to examine the genomic distribution of differential methylation analyzing the overlap between studied regions and DMRs across various genomic features (Fig. 2). Our results showed that DMRs were significantly enriched in intergenic regions, CpG islands and promoters, while genic regions and introns showed comparatively lower coverage when compared to all studied regions (Chi-square test, p < 0.05). In addition, we examined methylation levels across different genomic regions, including CpG islands, genic regions, and transcription start sites. As expected, methylation patterns varied across these regions: genic regions showed a characteristic decrease around the TSS, consistent with active transcription; TSS regions exhibited hypomethylation; and CpG islands displayed typically lower methylation levels (Fig. S1).

**Figure 2:**
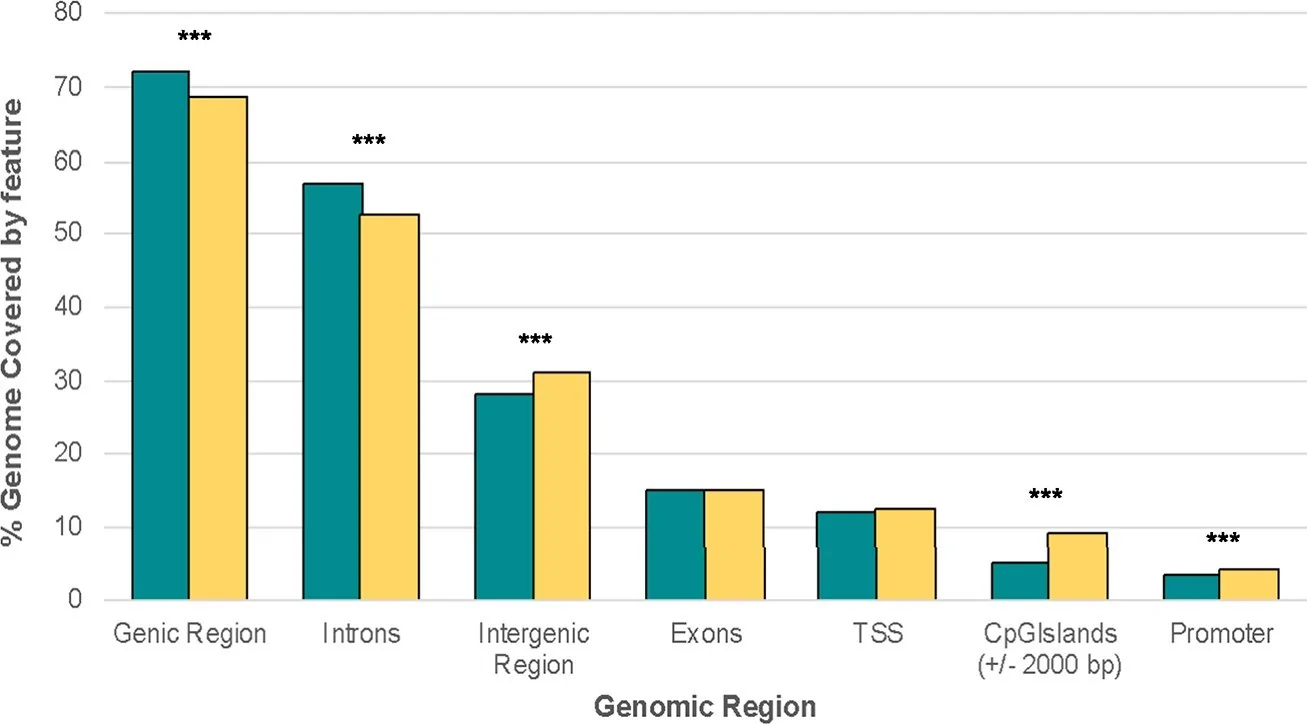
Localization of DMRs in different genomic regions. Green bars represent all studied genomic regions (tiles of 250 bp generated with a 125 bp sliding window) while yellow bars represent the significant DMRs (Q < 0.01). Chi-square test was applied to identify differences in the percentage of the genome covered by all studied genomic regions versus the DMRs (*P* < 0.05).

#### Methylation changes at the gene level: differentially methylated genes

To study methylation changes at the gene level, a more stringent statistic Q-value (Q < 0.001) was applied giving a total of 7 655 DMRs identified across all treatment groups (Ctrl vs 3 nM, Ctrl vs 30 nM, and 3 nM vs 30 nM). Our results showed that from those, 5 280 DMRs were located within genic regions (Supplementary Table 2A), with 4 309 located in gene bodies (Supplementary Table 2B), 1 100 in TSS regions (Supplementary Table 2C), and 370 in promoters (Supplementary Table 2D). Overall DNA methylation levels (0–100%) of genic DMRs showed that gene bodies were predominantly hypermethylated, whereas TSS regions and promoters were in general hypomethylated (Fig. S2). Note that the scale of Fig. S2 spans the full methylation range, which makes statistically significant differences between controls and TBT exposed fish not appear visually striking. Importantly, hierarchical clustering of analyzed samples clearly showed that all TBT exposed samples clustered separately from controls. Moreover, the vast majority of TBT samples at the same concentration also clustered together across all contexts (except one 30 nM sample for TSS that clustered with 3 nM TBT samples, and one 3 nM sample in the promoter regions that clustered with 30 nM TBT samples) confirming treatment-related patterns.

Subsequently, we examined the 5 280 DMRs located within genic regions corresponding to 3 066 unique differentially methylated genes (DMGs, Fig. 3). Similar to gene bodies, DNA methylation levels were predominantly hypermethylated, with a concentration-associated structure evident across replicates revealed by the hierarchical clustering, indicating consistent treatment-related patterns across samples (Fig. 3A). Interestingly, the protocadherin 1 gamma 1 (*pcdh1g1*), a gene predicted to enable calcium ion binding activity involved in cell adhesion and expressed mainly in the brain [25], contained up to 25 DMRs, and more than 100 genes contained 5 DMRs or more. To characterize the nature of the methylation responses, DMGs (identified as statistically significant across at least one of the treatment contrasts) were descriptively grouped into four patterns based on their average methylation trajectories across concentrations (Fig. 3B-C, Supplementary Table 2E): 1) patterns consistent with decreasing methylation across groups (n = 1174 DMGs); 2) patterns consistent with higher methylation at the intermediate concentration and lower values at control and high concentration (inverted-U shape pattern, n = 452 DMGs); 3) patterns consistent with lower methylation at the intermediate concentration and higher values at control and high concentration (U-shape pattern, n = 374 DMGs); 4) patterns consistent with increasing methylation across groups. Together, these results demonstrate that TBT exposure induces widespread, region-specific methylation changes, with different response patterns across conditions.

**Figure 3:**
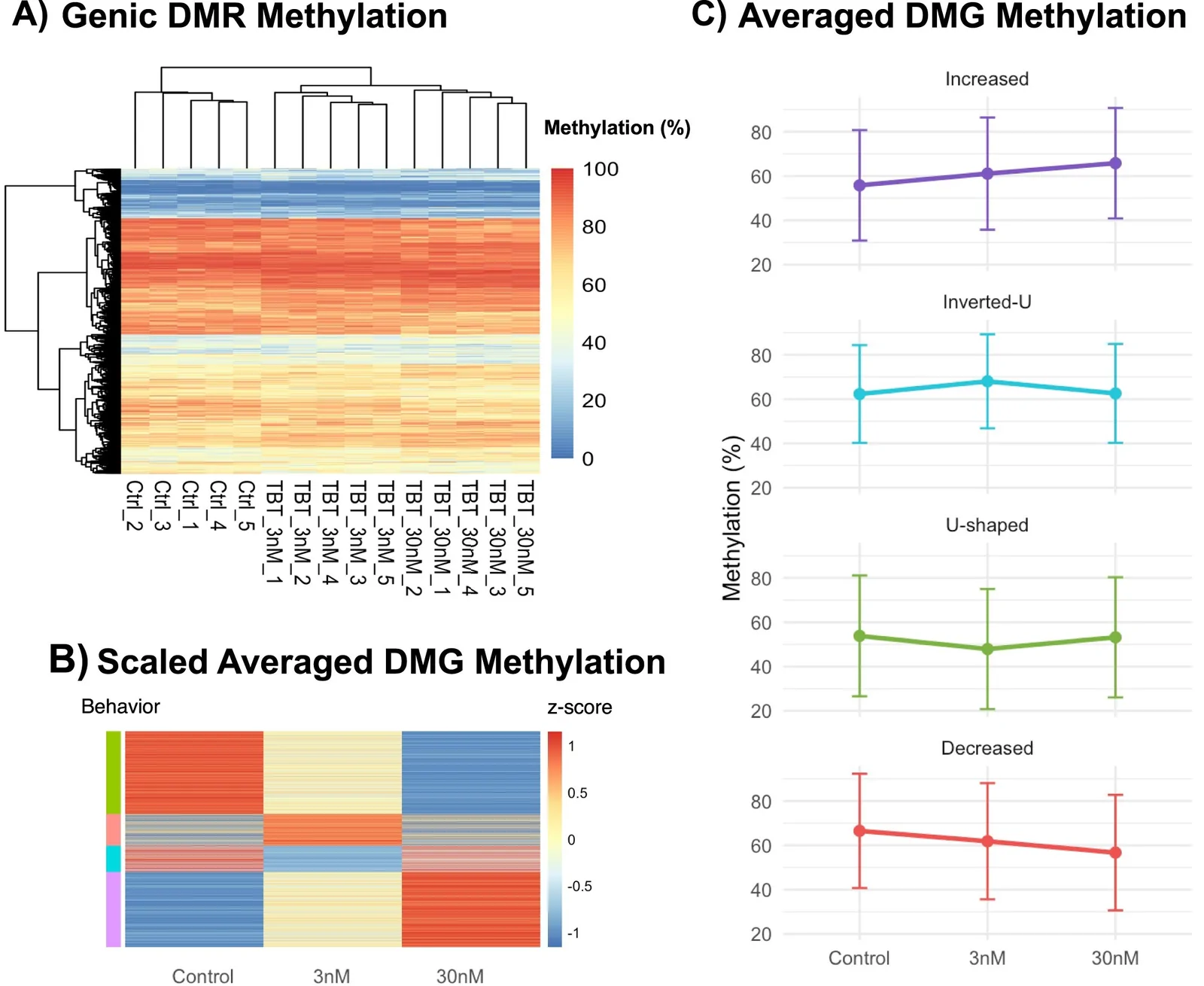
Characterization of DMRs within Genic regions (DMGs). (A) Heatmap plot showing methylation levels (%) for the 5280 DMRs located within genic regions. Bar colors represent DNA methylation levels (from 0–100%). Hierarchical clustering shows sample grouping by experimental group. (B) Scaled heatmap of averaged methylation values for the 3066 DMGs. Bar colors represent z-score-normalized methylation averaged values (row-scaled). DMGs descriptively grouped based on average methylation trajectories into four categories: 1) patterns consistent with increasing methylation across conditions; 2) Inverted-U shape, 3) U-shape, 4) patterns consistent with decreasing methylation across conditions. (C) Average methylation trajectories of all genes represented by each behaviour category across TBT concentrations. Error bars represent standard deviation. These plots illustrate descriptively observed monotonic (increasing/decreasing) and non-monotonic (U-shaped, inverted U-shaped) patterns across exposure conditions. Detailed annotations of DMRs located in genic regions and DMGs Supplementary Table 2A&E.

#### Functional enrichment analysis of DMGs

The previously identified 3 066 DMGs (derived from genic DMRs obtained after multiple comparisons across all treatments) were used downstream to perform GO and KEGG enrichment analysis (Supplementary Table 3). This analysis identified 246 altered biological processes (BPs) terms, 85 Molecular Functions (MFs) and 56 Cellular Components (CCs) that are summarized in Fig. 4. GO enrichment analysis highlighted significant biological processes included developmental processes such as anatomical structure development, nervous system development and embryonic morphogenesis. In addition, other pathways related to cell signaling, cellular organization, metabolic regulation, ion transport and localization and adhesion and cell movement were also found enriched. CC terms associated with DMGs included plasma membrane, synapse, presynapse, and GABA receptor complex, among others. Interestingly, for MF terms, DMGs were also significantly enriched in processes related to GABA receptor activity, in addition to semaphorin receptor activity, ligand-gated ion channel activity, and voltage-gated potassium channel activity, among others. Regarding KEGG analysis, nine KEGG pathways were identified as associated with DMGs, including cell adhesion molecules, the MAPK signaling pathway, Calcium signaling pathway, Adherens junction, Apelin signaling pathway, Hedgehog signaling pathway, ErbB signaling pathway, Notch signaling pathway and neuroactive ligand-receptor interactions (Fig. 5).

**Figure 4:**
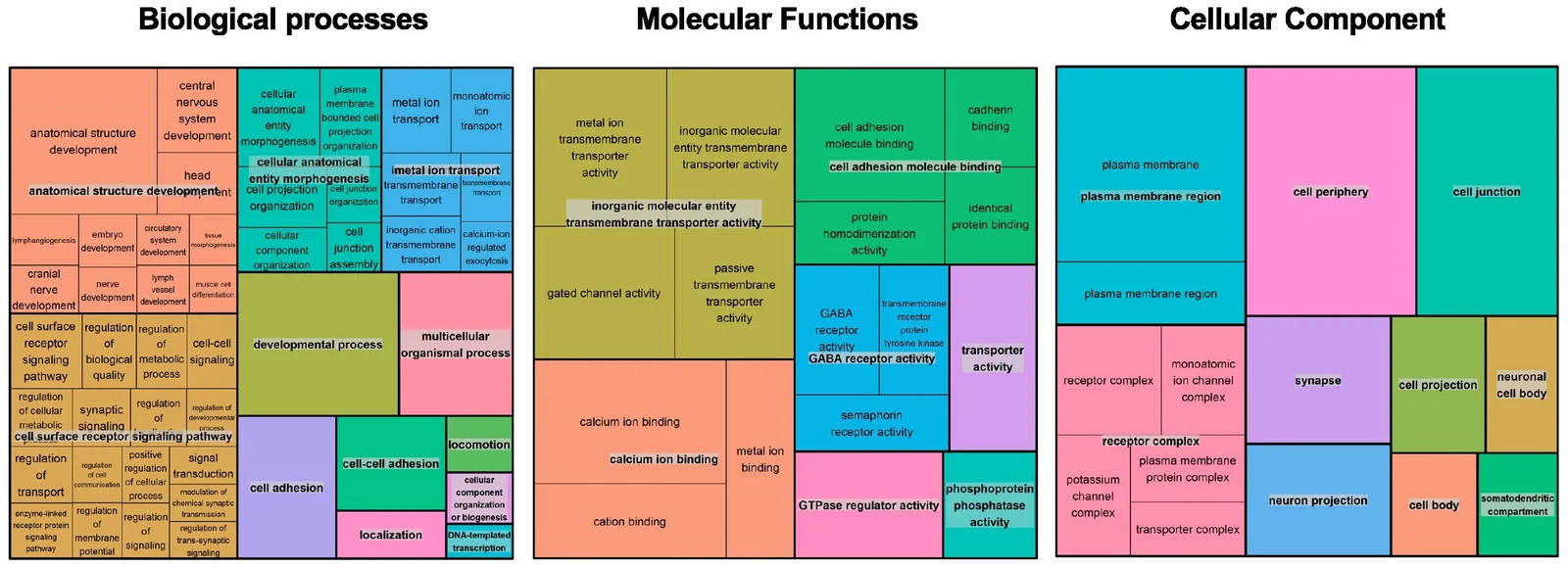
Gene Ontology enrichment analysis associated with TBT exposure. The analysis was performed using the 3066 DMGs in ShinyGO and summarized by a Revigo treemap where rectangle size reflects enrichment FDR values (not all terms are shown due to space limitations; see Supplementary Table 3).

**Figure 5:**
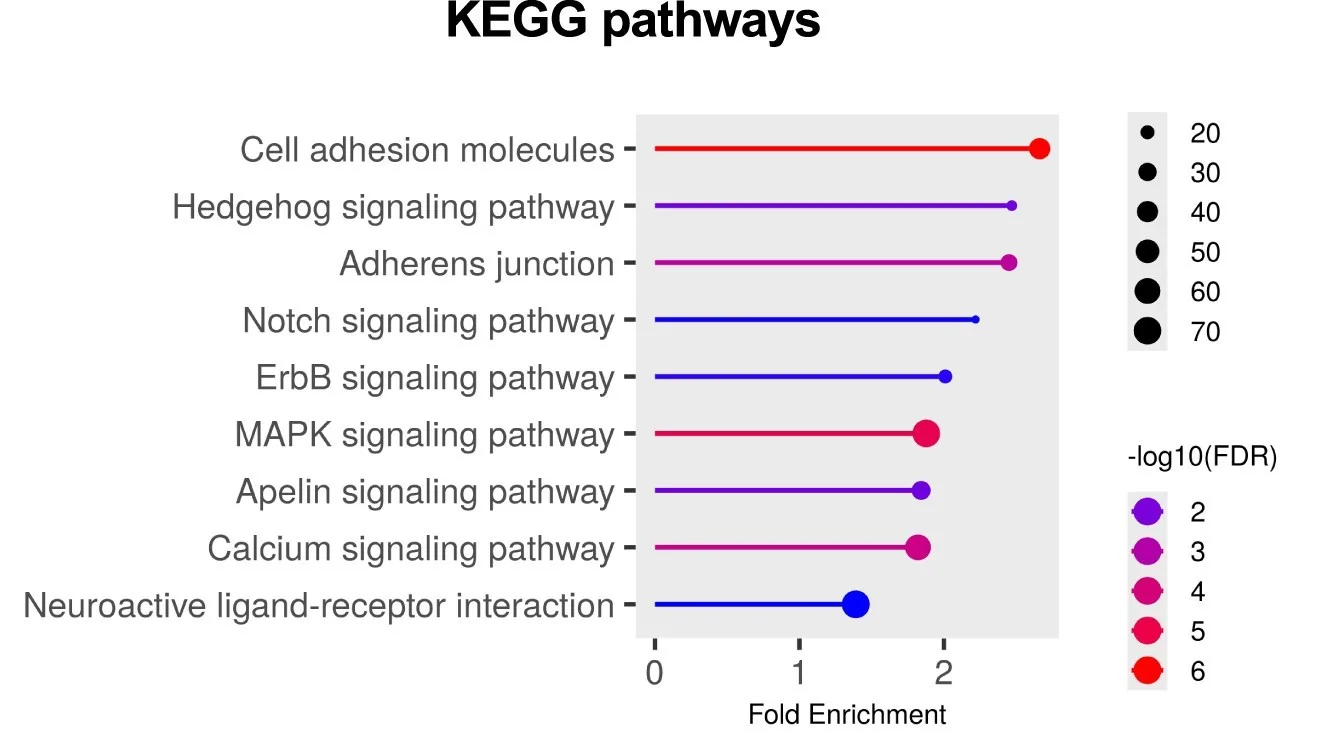
KEGG pathways enriched analysis associated with TBT exposure. The analysis was performed using the 3 066 DMGs in ShinyGO. Bubble size represents the number of genes in each pathway and colors represent statistical significance as FDR.

### Multi-Omics integration

#### Integration of RNA-Seq and WGBS: Identification of commonly affected genes and correlations

To investigate the relationship between differential methylation and gene expression changes induced by TBT, epigenomic analysis results were integrated with transcriptomic data from a previously published 2020 study [8] using data from 3 biological replicates that had DNA and RNA extracted simultaneously and finally analyzed (see materials and methods for more details). This integrative approach allowed for the assessment of whether differentially methylated genes had potential functional consequences on gene expression. Our results showed that out of 1 776 DEGs (Supplementary Table 4A) and 3 066 DMGs (Supplementary Table 4B), 292 genes were commonly affected at both mRNA and DNA methylation levels (Supplementary Table 4C). Notably, this overlap was found significant by both a hypergeometric test (p < 0.001) and Fisher’s exact test (p < 0.0001). The functional enrichment analysis revealed that those 292 commonly affected genes were involved in a total of 117 biological processes (BPs), 32 cellular components (CCs), and 15 molecular functions (MFs) (Fig. 6; Supplementary Table 4D). The most affected pathways were related to anatomical development, with main morphogenetic pathways altered, being consistent with previous the general developmental disruption observed in Martinez *et al*. [26]. Other relevant pathways altered were related to receptor localization to synapse, calcium ion binding, GABA signaling, GABA receptor activity and G protein couple receptor complex.

**Figure 6:**
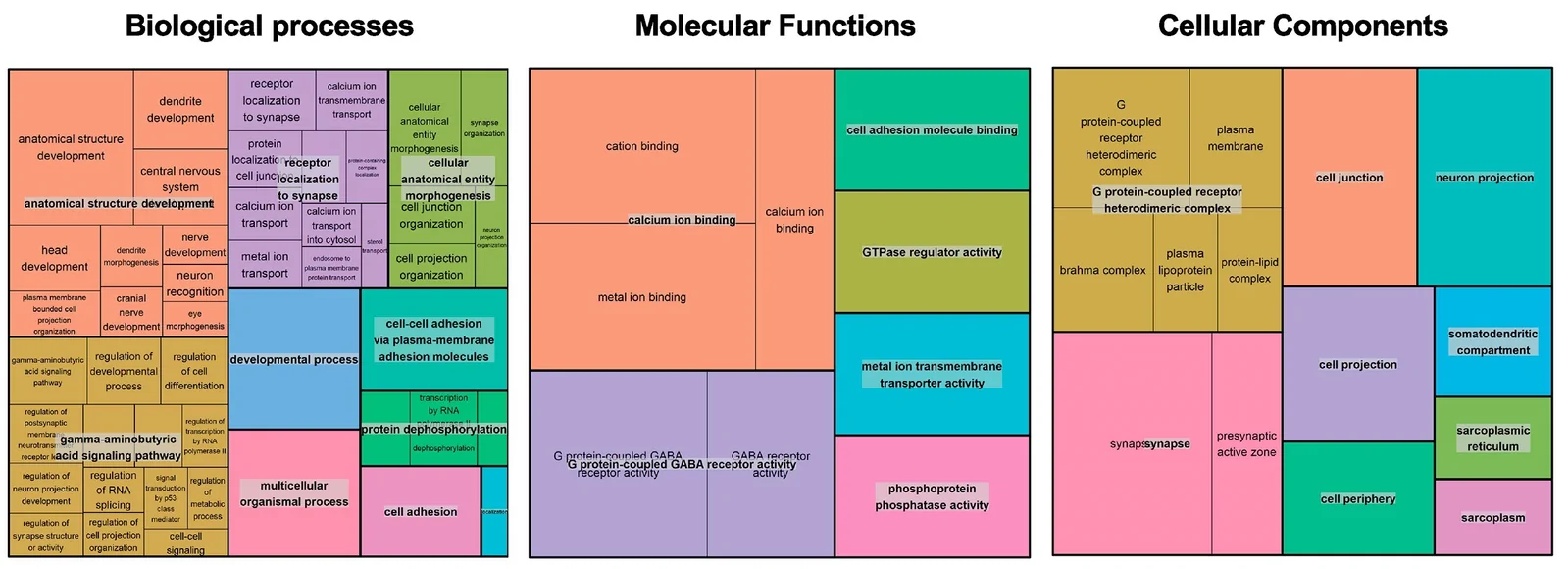
Gene Ontology enrichment analysis associated to genes commonly affected by TBT exposure at the transcriptomic and DNA methylation levels. The analysis was performed using the 292 common genes between differentially methylated genes (DMGs) and differentially expressed genes (DEGs). ShinyGO results were summarized by a Revigo treemap, where rectangle size reflects enrichment FDR values (not all terms are shown due to space limitations).

Additionally, correlation analysis between DNA methylation and gene expression was performed for 292 genes previously identified as showing changes in both features. Among the 292 genes analyzed, 28 displayed nominal Pearson correlations (p < 0.05), of which 16 showed positive and 12 negative associations between methylation levels and transcript abundance, exceeding the ∼14 correlations expected by chance (Fig. 7). A binomial test under the null hypothesis (p = 0.05) indicates that observing ≥ 28 nominal hits is unlikely by chance (P ≈ 8.5 × 10^-4^), suggesting enrichment of associations. However, after correcting for multiple comparisons using the Benjamini–Hochberg method, no correlations remained significant and should therefore be considered exploratory. We report correlation coefficients and confidence intervals to highlight effect sizes and directional trends, which may still provide biologically meaningful insights despite the lack of FDR significance (Supplementary Table 4E).

**Figure 7:**
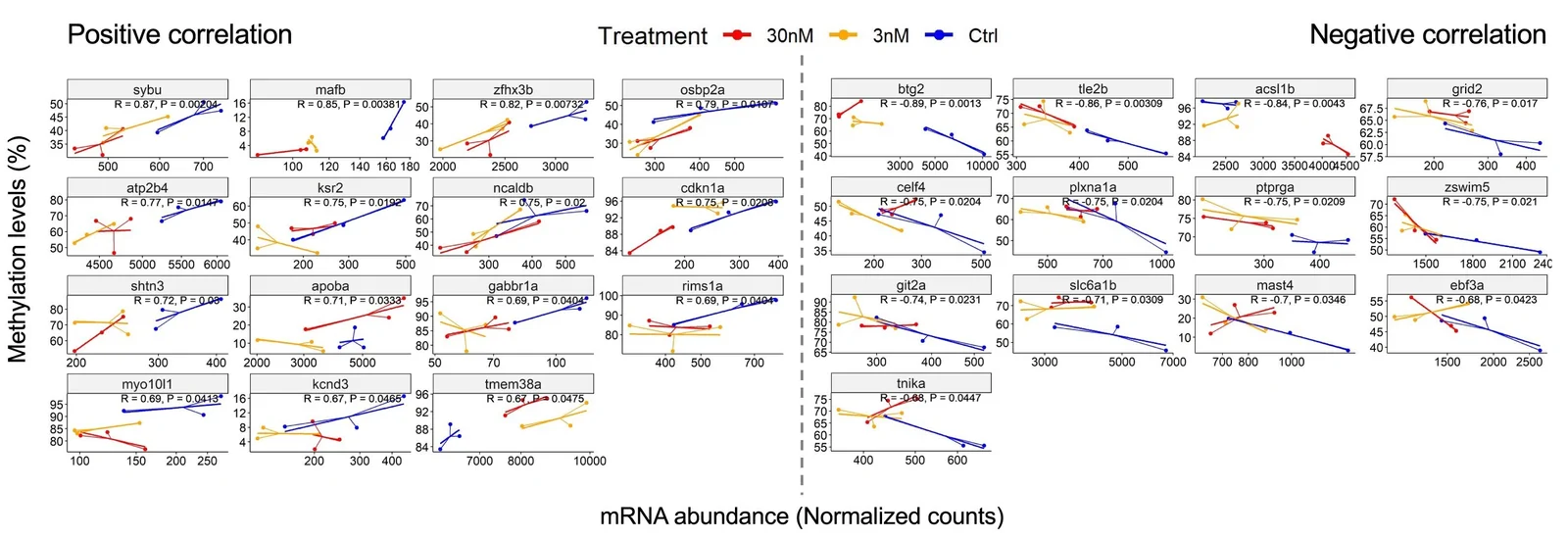
Gene-specific correlations between DNA methylation and mRNA abundance across all samples irrespective of the treatment group (treatment groups are shown only for visualization). Scatterplots display methylation levels (%) versus normalized mRNA counts for genes that presented both: (i) differential methylation and expression after single-omic analysis and (ii) nominally significant Pearson correlations (*P* ≤ 0.05). Genes are grouped by correlation direction: Positive correlation (left panels) and Negative correlation (right panels). Each panel includes the Pearson correlation coefficient (R) and *P*-value. Each dot represents DNA methylation and mRNA abundance of each sample, and the lines connect points of the same experimental group: blue (control), orange (3 nM) and red (30 nM). While several genes exhibit moderate-to-strong correlations, none remained significant after Benjamini–Hochberg correction for multiple testing. Results should be interpreted as exploratory, emphasizing effect sizes and trends rather than strict statistical significance.

#### Multi-Omics enrichment analysis

The multi-omic functional enrichment analysis using the PaintOmics 4 tool provided a comprehensive overview of the KEGG pathways altered by TBT exposure, considering three different omic levels: epigenomics, transcriptomics, and metabolomics. For each omic layer, KEGG pathway enrichment analyses were first performed independently using the corresponding sets of significantly altered features (DMGs, DEGs, and differentially regulated metabolites). The enriched pathways identified from each omic layer were then combined, resulting in a total of 181 KEGG pathways containing at least one significantly altered feature in at least one dataset. These pathways were subsequently used as input for joint multi-omic enrichment analysis in PaintOmics, which integrates information across omic layers at the pathway level.

Among the 181 KEGG pathways containing at least one significantly altered feature in any single omic layer, 45 pathways remained significant after joint multi–omic enrichment analysis in PaintOmics (Fig. 8A). PaintOmics illustrated a complex enrichment map, with several categories impacted. Among these, metabolism-related pathways were the most prominently affected, revealing disruptions in various biomolecules, including carbohydrates, lipids, proteins, and nucleotides. Notably, xenobiotic-related metabolism was found to be highly affected, being the pathways with the lowest joint p-value across the presented pathways (Fig. 8B).

**Figure 8:**
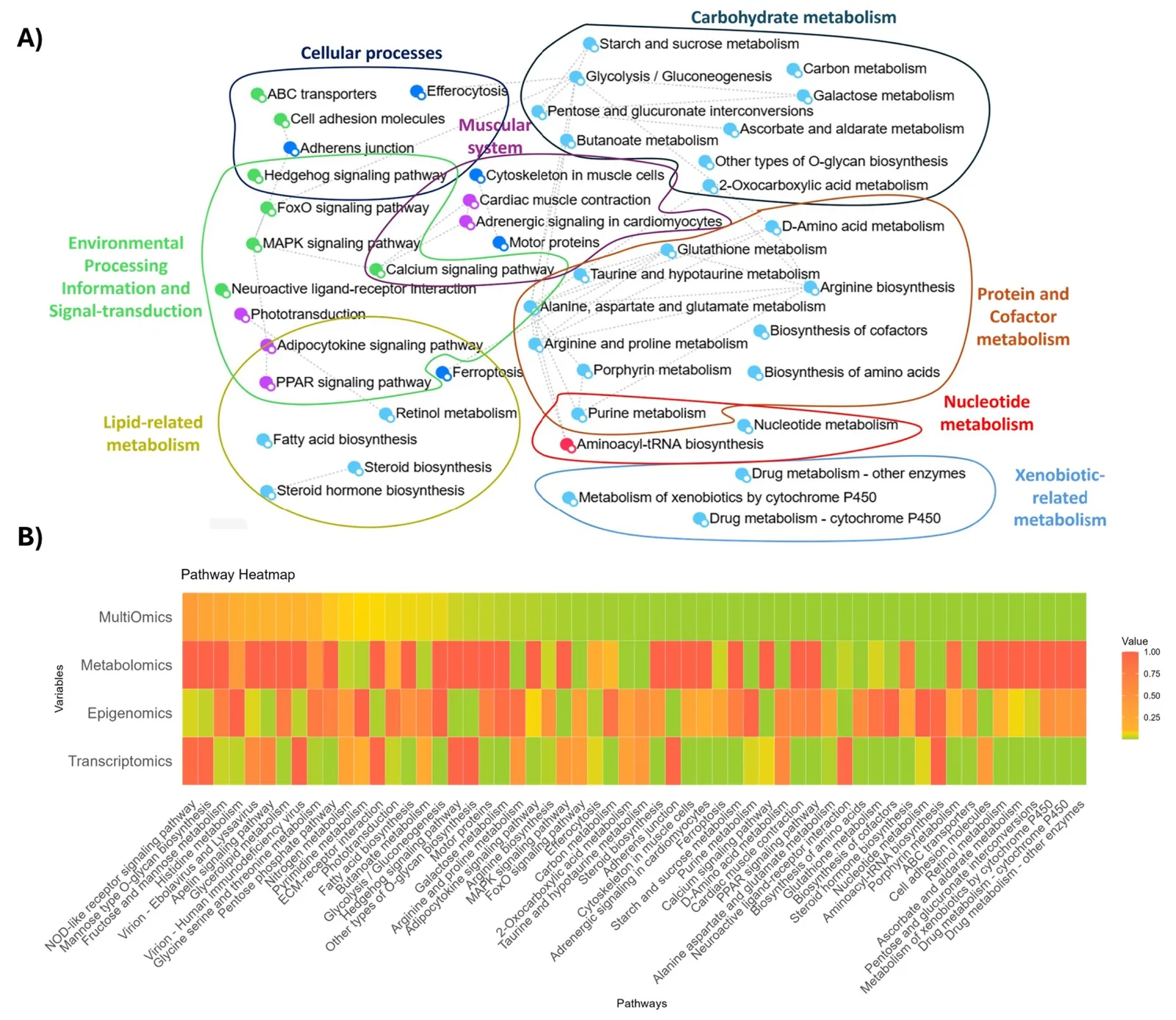
KEGG pathway enrichment analysis integrating epigenomics, transcriptomics, and metabolomics data to assess the effects of TBT exposure in zebrafish eleutheroembryos. (A) Pathway analysis performed in PaintOmics, displaying and grouping the significant pathways involved after TBT exposure. (B) Heatmap representing the *P*-values for significantly altered KEGG pathways across individual omics layers and the joint *P*-value combining all datasets. Green indicates pathways significantly enriched in a specific omic layer or in the combined analysis (*P* < 0.05).

Results obtained for the various significantly disrupted pathways highlight important differences across the studied omics, with 30 out of 48 significant pathways in the multi-omics analysis being significantly affected in only one omic layer. Epigenomics exhibited the most distinct and independent effects, while transcriptomics and metabolomics shared disruptions across approximately ten common KEGG pathways. Importantly, the only pathway found to be altered in both epigenomics, and metabolomics was related to neurotoxicity due to disruptions in neurotransmitter levels (neuroactive ligand-receptor interaction). However, it is important to emphasize that multi-omic analysis contributes to achieving more robust results. Some specific pathways identified as relevant in a particular omic analysis were found to be irrelevant when considering the overall joint p-value. For example, 17 relevant pathways identified in individual omic analyses—such as glycerolipids metabolism (transcriptomics), ECM-receptor interaction (epigenomics), and cholesterol metabolism (metabolomics) were significantly altered KEGG pathways when considering just one omic. However, their minimal significance in the other analyzed omics result in a joint p-value below the established significance threshold.

### Behavioural responses were altered following exposure to TBT

Zebrafish 5 and 8 dpf eleutheroembryos/larvae were challenged to two different types of stimuli to evaluate their reactive responses and cognitive capacities following early life exposure to relevant TBT concentrations. Three independent experiments were performed to ensure robustness of the results (n = 48 animals per condition and experiment). Three behaviour responses were assessed: the response to visual stimuli by the sudden absence of light (VMR), the escape response (startle response) evoked by a vibrational acoustic stimulus, and short-term habituation to a series of vibrational stimuli, a simple form of non-associative learning (Fig. 9). All responses assessed immediately following exposure, in 5 dpf eleutheroembryos, were significantly altered (VMR: Kruskal-Wallis H(2) = 9.586, *P* = 0.0083; Startle: H(2) = 25.76, *P* < 0.0001 and Habituation: H(2) = 43.73, *P* < 0.001). The visual response exhibited a non-monotonic (inverted U-shape) profile. Multiple comparison tests revealed statistical differences only between TBT 30 nM and 3 nM, where larvae exposed to the latter concentration reacted more strongly to sudden darkness while the reaction was significantly reduced in those exposed to the former (Fig. 9A). The startle response was significantly increased at the highest concentration of TBT (30 nM) (Fig. 9B) while no changes were observed for 3 nM. Following exposure, 5 dpf eleutheroembryos were transferred to a clean medium for a further three days, and behaviour responses were re-evaluated at 8 dpf. No statistical differences between groups were observed for the visual and the startle responses (Fig. 9D&E). On the other hand, the habituation profile remained similar to that observed for 5 dpf eleutheroembryos (Fig. 9F). Kruskal-Wallis test revealed significant differences among groups (H(2) = 30.39, *P* < 0.0001), which was mainly due to the considerable decrease of habituation time in larvae exposed to TBT 30 nM.

**Figure 9:**
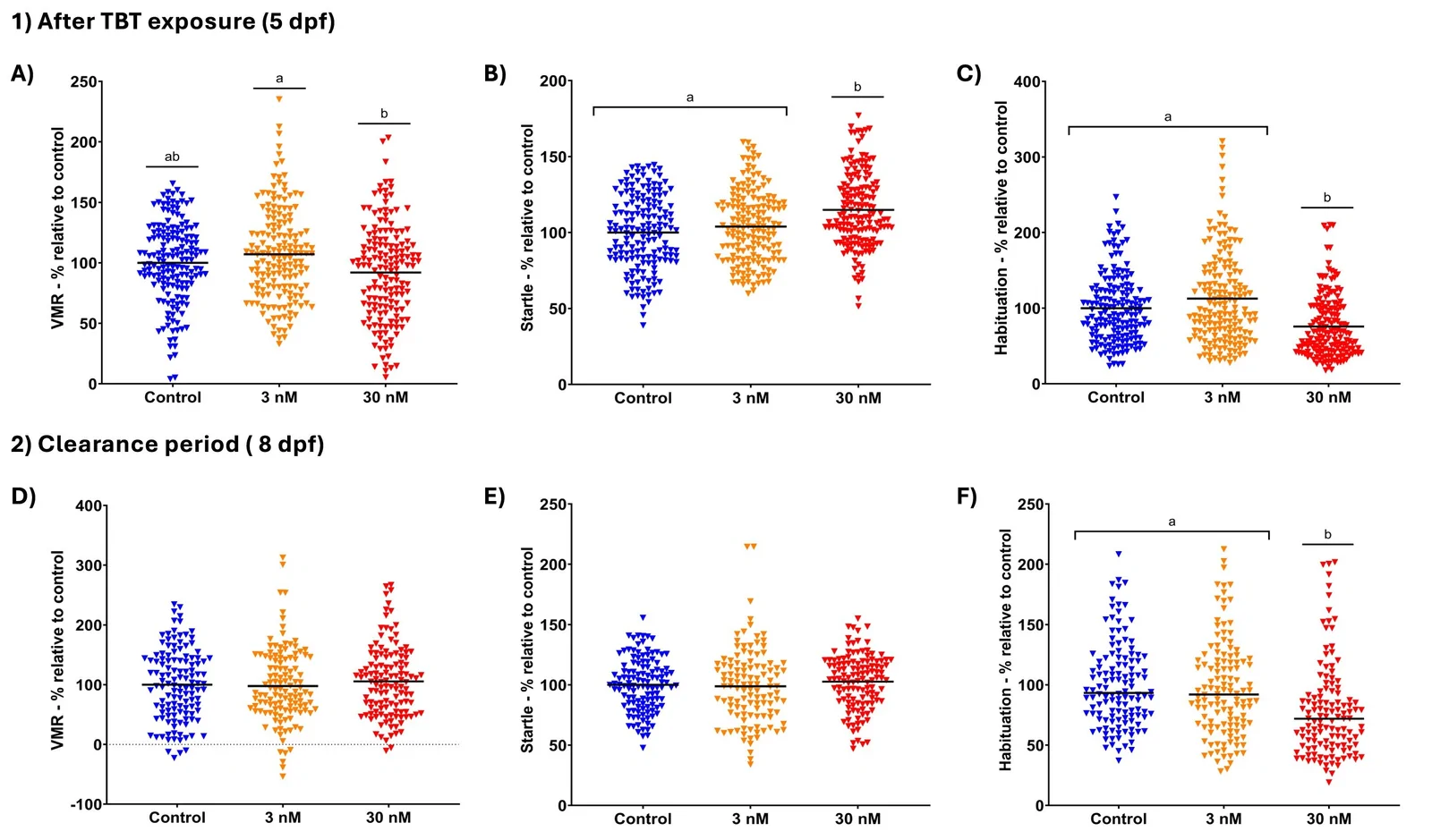
Behavioural changes on zebrafish at (1) five and (2) eight dpf eleutheroembryos/larvae. Fish were exposed for 72 h (from 2 to 5 dpf) to different TBT concentrations. A subset of animals was assessed for behaviour changes at 5 dpf and others subsequently transferred to clean water for 72 h (from to 5 to 8 dpf) and assessed later at 8 dpf. The results plotted are: A&D) Visual-motor response (VMR) (n = 68–84); B&E) Startle—acoustic/vibrational escape response (n = 79–82); and C&F) Habituation of the acoustic/vibrational escape response (n = 76–82). Data are represented in % relative to the control and are reported as scatter plots with the median (black line). Different letters indicate statistical subset groups following Dunn’s multiple comparison test (*P* < 0.01).

### Monoaminergic neurotransmitter profiles are altered by TBT developmental exposure

Monoaminergic neurotransmitters serotonin, dopamine, and norepinephrine, as well as their precursors, tryptophan, tyrosine, and L-DOPA, and their products, 5-hyroxyindolacetic acid (HIAA), 3,4-dihyroxyphenylacetic acid (DOPAC), 3-methoxytyramine (MT) and homovanillic acid (HVA) were determined in heads of control and TBT exposed zebrafish eleutheroembryos/larvae (6 biological replicates per condition, 15 heads/replicate; Fig. 10).

**Figure 10:**
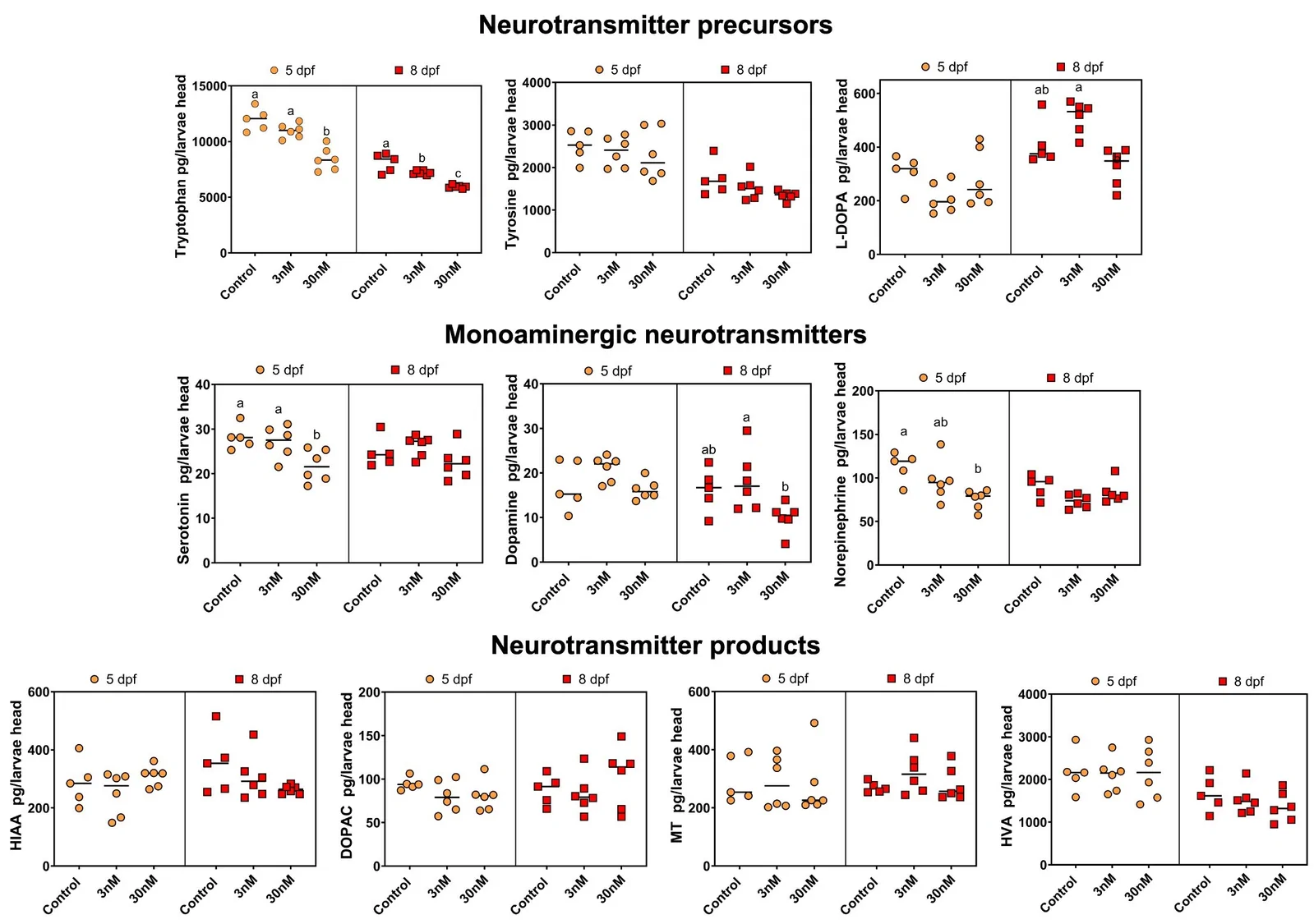
Neurotransmitter profiles of five and eight dpf zebrafish eleutheroembryos/larvae. Fish were exposed for 72 h (from 2 to 5 dpf) to different TBT concentrations. A subset of animals was assessed for neurotransmitter content changes at 5 dpf and others subsequently transferred to clean water for 72 h (from to 5 to 8 dpf) and assessed later at 8 dpf. Results are depicted as dotplots with the median (black line) and represented as pg/larvae head. Different letters represent different subset groups following the Tukey post hoc test (*P* < 0.05).

Levels of serotonin and its precursor tryptophan were found to be significantly decreased after TBT exposure at 5 dpf (one-way ANOVA: F (2,14) = 6.046, *P* = 0.0128; F (2,14) = 23.00, *P* < 0.0001, respectively). This decrease was also observed at 8 dpf, after a 3 days clearance period, but only in the case of tryptophan the decrease was found to be statistically significant (one-way ANOVA, F (2,14) = 29.90, and *P* < 0.0001). Early developmental exposure to TBT also significantly altered some catecholamine neurotransmitters levels following exposure and the clearance period. Tyrosine levels in both 5 and 8 dpf eleutheroembryos/larvae were not significantly altered by TBT exposure. Interestingly, L-DOPA and dopamine levels showed a U-shaped response with significant decreases respect controls were observed at 8 dpf larvae (one-way ANOVA, (F (2,14) = 10.37, *P* = 0.0017) and (F (2,14) = 4.173, *P* = 0.0379). The decrease in dopamine levels was more pronounced at 30 nM than that of L-DOPA. Norepinephrine levels were only altered in 5 dpf eleutheroembryos, showing a decrease across the tested exposure conditions (one-way ANOVA, F (2,14) = 6.175, *P* = 0.0120).

## Discussion

This study generated new whole–genome bisulfite sequencing (WGBS) data to investigate tributyltin (TBT)–induced neurodevelopmental disruption in zebrafish embryos during organogenesis. These methylation data were integrated with previously published transcriptomic and metabolomic datasets derived from the same exposure experiment and egg batch, and with newly generated behavioural and neurotransmitter endpoints obtained from independent follow–up experiments. The aim was to characterize epigenetic alterations across TBT concentrations and to integrate these molecular changes with functional endpoints. By adopting a multi-omic framework, we intended to provide a more comprehensive understanding of how early-life exposure to environmental contaminants can disrupt developmental pathways and inform future strategies for integrative risk assessment.

While previous research has highlighted the endocrine-disrupting and obesogenic properties of TBT, its effects on DNA methylation have remained largely unexplored [16]. Past epigenomic assessments on TBT effects have been more focused on transgenerational effects rather than DNA methylation [27]. Earlier studies have explored the effects of TBT on DNA methylation primarily through *in vitro* approaches. For instance, Sales *et al.*, demonstrated that TBT exposure leads to an increase in global DNA methylation levels in 3T3-L1 preadipocyte cells [28], although global assays lack locus-specific resolution at individual CpG sites. More recent research shifted towards high-throughput and locus-specific approaches to capture the complexity of epigenetic regulation. A field study in *Hexaplex trunculus* populations from highly TBT-polluted Adriatic sites showed up to 20-fold higher tissue TBT concentrations, increased imposex and body mass, and genome-wide hypomethylation, with epigenetic differentiation exceeding genetic differentiation, indicating pollution-driven epigenetic shifts linked to impaired reproductive fitness [17]. Collectively, these studies underscore the need for whole-organism, high-resolution approaches to probe TBT-associated epigenomic changes. Using WGBS, we observed widespread DNA methylation differences associated with TBT exposure. Our results identified widespread DNA methylation differences across TBT concentrations, with the majority of the DMGs (2 240) showing monotonic response patterns characterized by increasing or decreasing methylation levels across exposure groups. Interestingly, 826 DMGs displayed non-monotonic response patterns, highlighting the complexity of epigenetic regulation under endocrine-disrupting chemical exposure. Enrichment analysis of all DMGs showed over-representation of terms related to nervous system development, ion transmembrane transport, cell adhesion, ion transport and metabolic regulation. These findings add to growing evidence that endocrine-disrupting chemicals can modulate epigenetic pathways with potential developmental and metabolic consequences [76, 77, 78], providing mechanistic insights beyond traditional endpoints [29].

Integration of methylome, transcriptome and metabolome data revealed convergent signatures in neurochemical pathways, pointing to coordinated molecular changes under TBT exposure. Metabolomic analyses were performed on parallel pools of eleutheroembryos originating from the same exposure experiment and egg batch as the epigenomic and transcriptomic analyses, but not from the same individual pools; accordingly, multi–omic integration was conducted at the condition and pathway level rather than through direct one–to–one sample matching. Because the multi-omic datasets analyzed in this study derive from whole-embryo samples, the results should be interpreted as hypothesis-generating, as observed molecular changes may reflect shifts in tissue composition or cell-type proportions rather than uniform regulation across all cells. In addition, the use of pooled eleutheroembryos may mask inter–individual variability across epigenomic, transcriptomic and metabolomic responses. Consequently, the identified molecular alterations represent average population–level patterns rather than individual–specific responses. Importantly, the value of this multi–omic approach lies not in the identification of entirely novel pathways, many of which have been reported previously using single–omic strategies, but in the ability to prioritize pathways and candidate genes that show consistent support across molecular layers and functional endpoints. By requiring concordance between epigenomic, transcriptomic and metabolic alterations, and further anchoring these changes to neurotransmitter profiles and behaviour, the multi–omic framework increases robustness, reduces false–positive pathway associations, and strengthens biological plausibility. Exploring these cross-layer correlations provides a valuable foundation for generating prioritized hypotheses that can guide targeted validation and mechanistic studies.

Our data suggested that TBT exposure could be associated with neurotoxic perturbations, particularly in pathways related to neuroactive ligand-receptor interactions that regulate synaptic function (Fig. 11). These pathways involve a collection of receptors and ligands on the plasma membrane, mediating intracellular and extracellular signaling processes [30]. Disruptions were observed in amino acid biosynthetic pathways (including alanine, aspartate, and glutamate metabolism), primarily detected through metabolomics and transcriptomics, with minor contributions from methylomics. These pathways are critical because several neurotransmitters are directly derived from these amino acids, linking metabolic alterations to potential changes in neurotransmitter synthesis and signaling. In line with this, single-omics analysis showed that TBT exposure disrupted glutamate levels [10], a key precursor of GABA, alongside with transcriptomic changes in GABA-related genes [8]. Our study also showed changes in DNA methylation in genes encoding GABA receptor subunits (*gabbr1a, gabbr1b, gabrg2, gabbr2*). Notably, *gabbr1a* showed a positive correlation between DNA methylation and mRNA abundance, suggesting coordinated regulation at multiple molecular layers. In addition to GABAergic components, monoaminergic neurotransmitter analysis confirmed that TBT exposure disrupted dopaminergic and serotonergic systems, which are known to interact with GABA signaling and influence synaptic balance [31–33]. Several studies have also emphasized neuroendocrine systems as critical targets of EDCs [34, 35].

**Figure 11:**
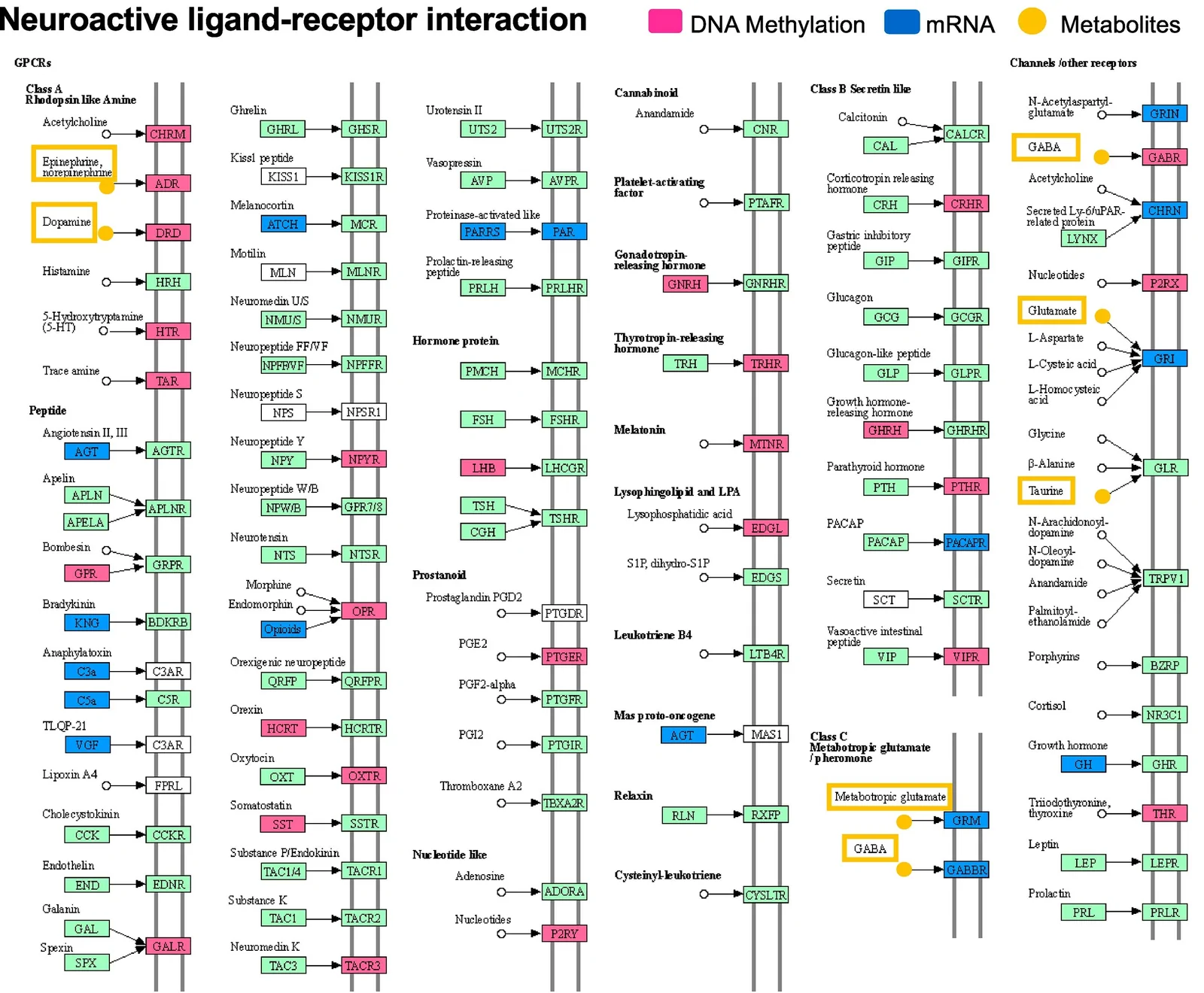
Summary of multi-omic (epigenomic, transcriptomic and metabolomic) alterations in the neuroactive ligand-receptor interaction pathway (KEGG Path: dre04080).

The observed multi-omic disruptions in neurotransmitter-related pathways, particularly those involving GABAergic and monoaminergic systems, raised the question of whether these molecular changes could be reflected at the organismal level. To address this, behavioural endpoints were assessed as organism-level indicators of neuroactive effects. Behavioural responses were consistent with these molecular findings, primarily at the highest concentration tested (30 nM), where acoustic startle response (ASR), visual motor response (VMR), and habituation were significantly altered. These endpoints are widely recognized as sensitive measures of sensorimotor and cognitive function in zebrafish and have been previously reported to respond to neuroactive compounds [40, 41]. Our results align with previous reports of TBT-induced behavioural changes in zebrafish and other fish species [36–38], suggesting that early-life exposure can influence sensorimotor and cognitive functions. For instance, TBT has been shown to significantly disrupt zebrafish eleutheroembryos’ behaviour at lower concentrations than the ones tested herein. Liang et al. [39], reported significant reductions in locomotor activity in 4 dpf zebrafish eleutheroembryos exposed to 1 nM TBT, indicating sensitivity at even lower concentrations.

Interestingly, the observed neurobehavioural effects (increased ASR and enhanced short-term habituation) aligned with the multi-omics data revealing epigenetic and transcriptomic alterations in GABA receptor subunits. Pharmacological studies demonstrate that modulating GABA receptors can bidirectionally influence these endpoints: GABA-C agonism and positive allosteric modulation of GABA-A receptors enhance short-term habituation to ASR in 5 dpf embryos, whereas GABA-A antagonism reduces habituation [40]. Likewise, GABA-A receptor agonists (such as propofol and etomidate) increase ASR magnitude, likely through paradoxical excitation [41]. Although direct causality cannot be established, the convergence of molecular signatures in neurotransmitter pathways with behavioural alterations provides correlative evidence linking early-life TBT exposure to neurofunctional disruption. We propose a hypothesis-driven follow-up to determine if epigenetic, transcriptomic and metabolic perturbations by TBT contribute to altered neural signaling to confirm mechanistic relationships. Also, it provides a strong basis for investigating how epigenetic regulation intersects with neurotransmitter homeostasis, while recognizing that mechanistic understanding will require tissue-specific and functional validation.

It is also worth noting that neurotransmitter and behavioural analyses highlighted the persistence of some TBT-induced alterations even after a clearance period. Although neurotransmitter profiles and behavioural responses showed slight improvement, significant effects were still observed, such as persistent cognitive dysfunction in the habituation response and changes in tryptophan, L-DOPA, and dopamine levels. This observation is consistent with previous reports of age-dependent morphological and behavioural changes following transient TBT exposure in zebrafish larvae [42] and with long-term neurobehavioural changes (including increased anxiety and fear and decreased aggression) in zebrafish juveniles that were extended into the next generation [16].

Beyond neurotransmitter-related pathway-level signals, integrated analysis identified candidate genes that may contribute to neurodevelopmental and behavioural outcomes. Among the genes the BTG antiproliferator factor 2 (*btg2*) emerged as noteworthy due to established roles in neuronal differentiation and growth regulation and reported sensitivity to endocrine signaling. In humans, BTG2 is known for antiproliferative properties and is implicated in DNA–binding transcription activator activity and RNA polymerase II–specific pathways, contributing to gene expression control and the growth arrest/differentiation of neuronal precursors [43]. In zebrafish, *btg2* has been predicted to participate in the negative regulation of cell population proliferation and is expressed in brain, hindbrain neural plate, and neural tube. We observed *btg2* hypermethylation accompanied by decreased transcript abundance in TBT-exposed eleutheroembryos, consistent with epigenetic repression. Prior work in adult zebrafish brains documented concentration-associated *btg2* suppression following TBT exposure and linked this to reduced estrogen (E2) and downregulation of *cyp19a1b* (brain aromatase) [38]. TBT is known to competitively inhibit aromatase, reducing circulating and brain estrogens, which play neuroprotective roles in synaptic health [44]. In vertebrates *btg2* has been implicated in aggression regulation via estrogen pathways in vertebrates [45, 46]. Interestingly, TBT-exposed zebrafish have been shown to display heightened aggression and fear responses (Braun, 2016). Aggressive behaviour was not assessed in our study, due to the limited maturation of social and aggressive responses of eleutheroembryos at 5 dpf, which impedes reliable assessment of these behaviours at this developmental stage. Other studies have linked *btg2* to neuronal resilience—including roles in protection from oxidative stress and apoptosis [47, 48]. In this regard, our multi–omic analysis indicated alterations in FoxO signaling (associated with oxidative stress resistance and apoptosis) and MAPK signaling (central to transducing extracellular signals into cellular responses), further supporting *btg2* as a plausible integration point. This positions *btg2* as a candidate environmentally responsive gene under TBT exposure, linking epigenetic regulation to neurodevelopmental transcriptional programs and behavioural outcomes.

The multi-omic approach also highlighted the well-established role of this organotin compound as an RXR signaling pathway agonist. RXR forms obligate heterodimers with multiple nuclear receptors (RAR, PPAR, PXR, and CAR) modulating diverse physiological processes. RXR partners with RARs to regulate retinol metabolism and embryonic development, with PPARs to control fatty acid metabolism and adipogenesis, and with xenobiotic sensors such as PXR and CAR to mediate the expression of genes involved in steroid metabolism and detoxification [49, 50]. Consistent with these interactions, our results revealed perturbation of pathways under RXR-containing heterodimers, notably retinol metabolism (RAR/RXR), steroid biosynthesis (PXR/RXR and CAR/RXR), and fatty acid biosynthesis (PPAR/RXR), across the tested TBT concentrations. Alteration of steroid and sterol hormones is particularly relevant given their critical roles in neurodevelopment [51]. Furthermore, our findings align with the classification of TBT as an obesogenic compound capable of dysregulating lipid-related metabolic pathways across species [52, 53]. For example, in *Daphnia magna*, TBT exposure induced glycerolipid accumulation via the glycerol-3-phosphate pathway [54, 55]. Notably, PPAR signaling disruption was evident only through multi-omic integration, not by single-omic analysis, reinforcing the value of integrative approaches. PPAR transcriptional changes influenced by RXR signaling have been associated with reproductive impairment, one of TBT’s hallmark toxic characteristics [56]. The epigenetic–transcriptomic correlation analysis, showed an inverse relationship in *acsl1b*, a gene involved in fatty acid metabolism and PPAR signaling. Although this association may be coincidental, the biological relevance of ACSL1 in lipid homeostasis and its integration within RXR/PPAR-regulated pathways makes it a plausible candidate for further investigation. In humans, ACSL1 has been linked to reduced fatty acid β-oxidation and increased triglyceride levels [57], suggesting conserved roles in lipid regulation. Given the established interaction between TBT and RXR signaling, *acsl1b* may represent an additional mechanistic key gene connecting epigenetic modulation to metabolic disruption, which requires validation in future studies.

In addition to neurotransmitter-related pathways, multi-omic integration highlighted enrichment in visual system processes, including phototransduction and retinol metabolism, primarily at the transcriptomic level. These findings are consistent with previous reports indicating that TBT can target retinal development and function. For example, a study demonstrated that TBT exposure induces apoptosis in retinal neuronal cells of zebrafish eleutheroembryos [58], while another study reported behavioural abnormalities in Japanese medaka juveniles, including altered proximity and movement patterns, which were linked to visual and neurological impairment [59]. Although our whole-embryo approach cannot resolve tissue-specific effects, these observations suggest that visual pathways may represent an additional sensitive target of TBT toxicity.

Beyond gene-specific observations, our findings also revealed broader epigenomic patterns and interpretative challenges that warrant consideration. Observed changes in DNA methylation caused by TBT were also linked to regulatory regions such as intergenic regions, CpG islands, and promoters. This suggests that TBT may perturb gene regulatory mechanisms by modifying epigenetic landscapes in regions essential for transcriptional control, thereby influencing genome function. However, regulatory elements within intergenic regions remain largely uncharacterized, limiting the ability to connect these changes to specific gene regulation. Furthermore, only a small fraction of genes showing DNA methylation changes also exhibited differential expression, and among these, just a subset displayed a significant correlation between methylation status and transcript abundance. This lack of directional correlation is a common challenge in environmental epigenetic studies and reflects several factors: (i) the dynamic nature of both the epigenome and transcriptome; (ii) transcriptional regulation mediated by multiple mechanisms beyond methylation, including histone modifications and non-coding RNAs; (iii) the contribution of trans-acting mechanisms, such as changes in transcription factor activity or signaling pathways; and (iv) tissue and cell specificity, which cannot be resolved in whole-embryo analyses [60]. Within this context, overinterpretation of isolated molecular correlations as causal relationships should be avoided.

To advance integrative risk assessment, future studies should incorporate multiple time points, higher biological replication, and expanded exposure designs to distinguish transient from persistent responses and improve mechanistic inference. In addition, current omic approaches relying on whole-body analyses, such as those used in this study, face significant limitations because they mask cell-type and tissue-specific responses. To overcome this, future research should incorporate advanced methods that preserve cellular and spatial resolution. Single-cell methylome and transcriptome sequencing (scMT-seq), simultaneously profiles DNA methylation and gene expression at the individual cell level [79], while transgenic zebrafish lines with fluorescently labeled cell populations could enable tissue- or organ-specific analyses. In addition, state-of-the-art spatial multi-omic technologies can preserve the native distribution of biomolecules, providing critical context for linking chemical disruption to molecular mechanisms [61–63].

Integration of multi-omic layers offers a more comprehensive view of pollutant-induced perturbations than single-omic approaches, enabling the identification of coordinated molecular responses rather than isolated signals. However, this complexity demands robust statistical frameworks for data fusion, such as DIABLO [64] or MOFA [65], and pathway-based visualization tools such as PaintOmics to translate high-dimensional correlations into biologically meaningful insights. Purely mathematical correlation metrics in high-dimensional data are constrained by sample size, noise, and multicollinearity. Therefore, moving toward causal and biologically grounded frameworks, aligned with Adverse Outcome Pathways (AOPs) and systems-level models, will improve inference. As costs decline, systematic multi-omic designs will progressively transform molecular ecotoxicology from observational studies into predictive frameworks capable of linking molecular perturbations to ecological outcomes. However, multi-omic data integration workflows still face technical and computational challenges, including data heterogeneity across omic layers, differences in dynamic range and feature dimensionality, missing values, batch effects, and limitations in statistical power for robust cross–layer integration [22]. Finally, future research should move beyond single-compound exposures toward mixture toxicity assessments aligned with the exposome concept. The exposome framework promotes mixture analyses coupled with omics to disentangle cumulative or synergistic effects [66, 67].

Although multi–omic approaches require greater experimental and analytical effort than single–omic designs, our results demonstrate that this added complexity yields a qualitative advance by filtering pathway–level signals, identifying cross–layer convergence, and linking molecular perturbations to organism–level outcomes. In this context, multi–omics should be viewed as a hypothesis–prioritization strategy rather than a replacement for targeted mechanistic studies, providing a rational basis for selecting pathways and genes for downstream validation. Our findings illustrate that differences can be detected across multiple biological layers—from epigenetic marks to behavioural endpoints—providing complementary perspectives on chemical impact. Importantly, these layers do not necessarily overlap, which is expected in whole-embryo approaches and reflects the complexity of organismal responses. Rather than being a limitation, this multi-layered view offers a framework for future risk assessment strategies. For example, targeted panels focusing on CpG islands and first introns, combined with transcriptomic and phenotypic screening, could serve as early indicators of neuroactive or endocrine-disrupting potential. Integrating all these complementary approaches into tiered testing pipelines will shift molecular ecotoxicology from descriptive observations toward predictive, mechanism-informed assessments capable of linking molecular perturbations to organismal and ecological outcomes.

## Conclusions

Together, our findings provide a high–resolution view of TBT–induced methylome alterations and demonstrate how integrating epigenomic data with transcriptomic, metabolomic, neurotransmitter and behavioural endpoints strengthens mechanistic inference in developmental neurotoxicity. Rather than relying on individual molecular layers, the multi–omic framework prioritizes pathways and candidate genes that show convergent support across biological scales, thereby increasing robustness and biological relevance. Persistent alterations in neurotransmitter profiles and behaviour following a clearance period further suggest that early–life epigenetic perturbations may contribute to lasting neurofunctional effects. By anchoring molecular changes to functional outcomes, this study illustrates how multi–omic strategies can move environmental epigenetics beyond pathway cataloguing toward hypothesis–driven, mechanism–informed risk assessment.

## Materials and methods

### Zebrafish rearing conditions and eleutheroembryo exposures

Adult zebrafish housing as well as eleutheroembryo exposures were performed as described by Martinez *et al*. [8]. Briefly, zebrafish eggs were obtained by natural mating of adult individuals (wild-type Danio rerio, 12–18 months old). The fertilization rate was at least 80%, and fertilized eggs collected at 2 hours post fertilization (hpf) were placed in 100 mL crystallizers with fish water (90 μg/mL of Instant Ocean water) at a density of 3 embryos/mL density. Eggs, eleutheroembryos and larvae were maintained under standard controlled conditions (28 ± 1°C, 12 L:12D photoperiods) during the experimental procedure.

TBT (CAS No. 1461–22-9; purchased from Sigma Aldrich (St. Louis, MO, USA)) stock solutions were prepared in dimethyl sulfoxide (DMSO) and stored at − 20°C. During the experimental period, fresh exposure solutions were prepared daily by diluting the stock solutions in fish water, obtaining a final DMSO concentration of 0.2% (v/v) and TBT concentrations were measured [8]. Nominal concentrations were chosen based on previous findings regarding survival, hatching, and swim bladder inflation rates [26]. The highest tested concentration in this study (30 nM) was previously reported as the no observable effect concentration (NOEC). Based on that, the experimental groups were as follows: control (0.2% DMSO), and TBT at 3 and 30 nM. Exposures were carried out with the positions of different experimental groups randomized on the plates to prevent potential bias.

Zebrafish eleutheroembryos were exposed between 2 and 5 days post–fertilization (dpf) to target a developmental window in which major morphological development has occurred, while neurodevelopmental processes and epigenetic programming remain highly active and sensitive to endocrine disruption. This exposure window was selected to minimize confounding effects of general developmental toxicity and enable the detection of neurodevelopment–specific and epigenetic perturbations. To confirm the absence of nonspecific developmental effects, embryo development was monitored daily using established morphological endpoints [68], including survival, hatching, and swim bladder inflation, with no differences observed between control and TBT–exposed groups across any parameter, indicating normal anatomical development across all exposure conditions [8].

As presented in Fig. 1, two independent experiments were conducted along this study. At the end of the exposure period for Batch 1, whole eleutheroembryos were collected, snap frozen in dry ice, and stored at -80°C for multi-omic analysis. In addition, a separated group of animals from a different clutch of eggs (Batch 2) were used for behavioural and neurotransmitter profile analysis. Assays and sample collection were conducted in a group of fish at the end of the exposures (5 dpf), while another group was transferred to clean fish water for 72 h (8 dpf), after which, behaviour assessment was conducted, and samples were collected and stored.

All experimental procedures were conducted under the license DAMM 7669, 7964 from the competent authority in accordance with the institutional guidelines. They were approved by the Institutional Animal Care and Use Committees of the Research and Development Centre (CID) of the Spanish National Research Council (CSIC).

### DNA extraction, library construction and high throughput sequencing

The use of pooled eleutheroembryos was selected as a compromise between achieving sufficient sequencing depth for whole–genome bisulfite sequencing, maintaining biological replication, and ensuring technical and economic feasibility. Simultaneous extraction of DNA and total RNA was performed from five independent pools of 10 eleutheroembryos per condition using the AllPrep DNA/RNA Mini Kit (Qiagen Iberia, Barcelona, Spain), following the manufacturer’s instructions. DNA and RNA quality and concentrations were assessed using a NanoDrop® 8000 UV–Vis Spectrophotometer (ThermoScientific, Bremen, Germany). In both cases, RNA and DNA libraries derived from high-quality samples, were sent to the National Center for Genomic Analysis (CNAG, Barcelona, Spain) for high-throughput sequencing. In the case of RNA-Seq analysis only three biological replicates were analyzed (already published and described in detail in Martinez *et al*. [8]), while for WGBS all five biological replicates were analyzed and data newly generated for this study.

Genomic DNA (1.0 μg) was spiked with unmethylated λ DNA (5 ng of λ DNA per μg of genomic DNA; Promega) and methylated T7 phage DNA (5 ng of T7 DNA per μg of genomic DNA), then sheared using a Covaris™ LE220-Plus (Covaris, Woburn, MA, USA). The fragmented DNA was size-selected to 150–300 bp using Beckman Coulter AMPure XP beads. Libraries were prepared using the KAPA HyperPrep Kit (Roche, Basilea, Switzerland). After adapter ligation with xGen™ Methyl UDI-UMI Adapters (Integrated DNA Technologies), the DNA was treated with sodium bisulfite using the EpiTect Bisulfite Kit (Qiagen Iberia, Barcelona, Spain). The libraries were sequenced on a NovaSeq 6000 (Illumina, San Diego, CA, USA) with a read length of 2 × 151 bp, following the manufacturer’s protocol for dual indexing. Image analysis, base calling, and quality scoring were performed using the manufacturer’s software, Real Time Analysis (RTA v3.4.4), followed by the generation of FASTQ sequence files. The sequencing data generated in this study have been deposited in the European Nucleotide Archive (ENA) and are freely accessible under the accession number PRJEB83100.

### Whole genome bisulfite sequencing data analysis

FASTQ files were quality-checked using FastQC (v0.11.7, Babraham Institute, UK), whereas adapter trimming was performed with Trim Galore (v0.6.4, Babraham Institute, UK) using standard parameters. Trimmed sequences were then mapped to the zebrafish genome (GRCz11) using Bismark (v0.22.3, Babraham Institute, UK) with the stringent option -L 30 and slightly relaxed mapping parameters (score-min L,0,-0.4), resulting in a mapping efficiency of approximately 74% (Supplementary Table 1A).

Differential methylation was assessed using the methylKit package (v1.16.1) in R (v4.0.3). Differentially methylated regions (DMRs) were identified using logistic regression with a sliding linear model (SLIM) to correct for multiple comparisons, applying Benjamini–Hochberg false discovery rate (FDR) correction (Q-value). To mitigate potential biases, a 99.9th percentile cut-off in read depth was applied in methylKit to exclude regions with extremely high read depths, which could result from PCR bias or mapping to repetitive regions. Tiles of 250 bp were generated with a 125 bp sliding window, and methylKit analysis was performed on tiles containing at least five mutually analyzed Cs with a minimum of five reads per C. These tiles were subsequently used to identify differentially methylated regions (DMRs). For each genomic window, methylKit compared methylation levels across all treatment groups (Ctrl, 3 nM, and 30 nM) using logistic regression. DMRs were defined as regions showing statistically significant methylation differences among groups (Ctrl vs 3 nM, Ctrl vs 30 nM, and 3 nM vs 30 nM) after multiple-testing correction (Q < 0.01).

First, the significant DMRs (Q < 0.01) were imported to the SeqMonk genome browser (v1.46) to investigate enrichment across different genomic features, including genic regions, intergenic regions, gene promoters, gene bodies, introns and exons, transcriptional start site (TSS) surroundings, and CpG islands. Genic regions were defined as gene bodies (exons and introns) ± 5 000 bp, while gene promoters were considered 1000 bp upstream of gene bodies. TSS regions were defined as ± 2 000 bp. CpG islands were identified based on the Takai and Jones algorithm (Takai and Jones, 2002). Due to the differences in GC content and the observed/expected (o/e) ratio of CpG sites in zebrafish compared to mammals, we adjusted the parameters (CG > 0.45, o/e > 0.65) and defined CpG shores as 2000-bp regions surrounding CpG islands. Chi-square test was applied to identify differences in the percentage of the genome covered in different genomic features by all studied genomic regions versus the DMRs (*P* < 0.05).

Further analyses were conducted using a more stringent approach, selecting significant DMRs with Q < 0.001. In this case, the SeqMonk genome browser (v1.46) was used to assign DMRs to differentially methylated genes (DMGs), which were then utilized for further downstream biological interpretation. For each gene, mean DNA methylation levels were calculated across the three treatment groups (Control, 3 nM, and 30 nM). DMGs were descriptively categorized based on average methylation values across conditions, with patterns interpreted as monotonic or non–monotonic without formal statistical trend testing. Subsequently, gene ontology (GO) and Kyoto Encyclopedia of Genes and Genomes (KEGG) pathway analyses were performed using ShinyGO (v0.81) (Xijin Ge, Jung and Yao, 2020). The significant GO terms were further visualized using the online tool REVIGO (v1.8.1) [69].

### Multi-omic analyses

In this study, a comprehensive characterization of TBT-induced disruption in zebrafish eleutheroembryos was achieved by combining epigenomic data with two distinct multi-omic approaches. First, an integrated analysis on epigenomic and transcriptomic data was performed from RNA and DNA extracted simultaneously from the same eleutheroembryo pools to identify potential correlations between DMRs and DEGs. Additionally, a pathway enrichment analysis incorporating metabolomic with epigenomic and transcriptomic data was conducted to enhance biological interpretation and uncover mechanistic insights more effectively.

#### Correlation analysis of DMRs and its gene expression

To investigate the relationship between DNA methylation and mRNA abundance, a correlation analysis was performed alongside statistical significance testing. The analysis integrated the DMGs identified in this study with the previously reported differentially expressed genes (DEGs) found by RNA-sequencing [8].

It is worth noting that since the previously published transcriptomic analysis (raw data GSE139599 from GEO repository), included a higher dose (100 nM) not included in the present study. Therefore, for the present study we only considered data obtained from the same samples analyzed by both studies. In addition, is worth noting that in the case of RNA-seq, we only analyzed 3 out of the 5 biological replicates available. Transcriptomic data was reanalyzed using DEseq2 (v1.46.0) [70].

Pearson’s correlation analyses were performed for genes identified as both differentially methylated and differentially expressed. Nominal p–values were initially used to assess potential associations (*P* < 0.05), and all correlations were subsequently adjusted for multiple testing using the Benjamini–Hochberg false discovery rate (FDR) correction. Correlation coefficients and directions were retained for exploratory interpretation, with effect sizes reported to support hypothesis generation rather than formal statistical inference.

#### Pathway enrichment analysis integrating epigenomics, transcriptomics and metabolomics

To elucidate the mechanism of action of TBT and gain a deeper understanding of its potential toxicological consequences, a novel multi-omic approach was implemented, integrating epigenomic, transcriptomic and metabolomics data. Metabolomic data was obtained from a previous study conducted also in our research group [10] housed in the Zenodo repository (ref. 8 297 064). In brief, metabolomic data was obtained from a subset of animals from Batch 1 (five independent biological replicates (20 eleutheroembryos/pool per condition).

For the multi-omic integration, DMGs, DEGs and differentially regulated metabolites (DRMs) that showed statistically significant changes in response to TBT exposures at 3 nM and 30 nM in the single-omics analysis were considered. To improve the graphical visualization and subsequent interpretation of biological data, PaintOmics 4, a web-based tool designed for the integrative visualization of multi-omic datasets within Kyoto Encyclopedia of Genes and Genomes (KEGG) pathway diagrams, was employed [71]. This approach relies on pathway enrichment analysis using Fisher’s Exact test, a robust method for understanding the biology underlying multi-omics data resulting from untargeted profiling technologies. Its fundamental principle involves grouping extensive lists of omic features into smaller sets of related biological features, typically organized by biological processes [72]. Specifically, PaintOmics 4 organizes the assorted genes and metabolites according to their participation in specific KEGG pathways. Significance levels were set to p < 0.05.

### Behavioural analysis

Behaviour assessment was conducted in all studied groups (control, 3 nM and 30 nM TBT) immediately at the end of exposures (5 dpf) and following the 72 h recovery period (8 dpf). In particular, a battery of three independent behaviour assays were conducted as described in Faria *et al.*, [73] in 5 and 8 dpf eleutheroembryos/larvae to measure: i) acoustic/vibrational escape response (startle), determined as the total distance (cm) traveled following the delivery of a sudden acoustic/vibrational stimulus; ii) habituation of the acoustic/vibrational escape response (non-association learning) evoked by a series of 50 tapping stimuli delivered every second calculated as the area under the curve (AUC); and iii) the visual motor response (VMR), reporting the response of larvae to a transition of light to dark and represented as the difference of the total distance (cm) traveled by larvae during 2 minutes after and before the transition of light to dark [73]. Larvae were placed in 48-well plates with one individual per well, containing 1mL of exposure medium. All trials were performed in the DanioVision Observation Chamber equipped with an IR-sensitive camera Basler acA1300-60gm (Basler Inc., Exton, PA, USA) and a temperature control unit (DanioVision, Noldus Information Technology, Leesburg, VA, USA) maintaining the temperature at 28°C. Videos were recorded at 30 frames per second under near-infrared light. Behavioural trials, video tracking, and analysis were conducted using the EthoVision XT 11 software (Noldus, Wageningen, The Netherlands). Three independent experiments were performed (n = 48 animals per condition in each experiment). To pool data from all experiments, results were transformed to % relative to the corresponding control mean value of each experiment. Statistical analysis and plots were accomplished using GraphPad Prism 10, where the analysis of variance was accessed using Kruskal-Wallis since data failed the normality distribution test (p < 0.05, Shapiro-Wilk) and specific differences between exposure groups were conducted using Dunn’s multiple comparison test.

### Neurotransmitter content analysis

The extraction procedure, as well as the chromatographic and mass spectrometry conditions to analyze targeted monoaminergic Dopamine, 3,4-dihydroxyphenylacetic acid (DOPAC), homovanilic acid (HVA), 3,4-dihydroxyphenylalanine (L-DOPA), serotonin, tryptophan, Tyrosine, 3-methoxytyramine (MT) 5-hydroxyindoleacetic acid (5-HIAA) and norepinephrine compounds were performed as described in previous studies addressing neurotransmitter content characterization in zebrafish larvae [74]. Briefly, pools of 15 heads of zebrafish larvae (n = 6 pools, per treatment including controls, 3 nM and 30 nM TBT), were collected after the behavioural assays at the end of the exposure period (5 dpf) and following the 72 h recovery period (8 dpf) and analyzed by LC-MS/MS [75]. More details about the analytical performance can be found in the Supplementary material. Data represented as pg/larvae head, were plotted and analyzed with GraphPad Prism 10. Data from all variables was found to have a normal distribution (p > 0.05, Shapiro-Wilk) and therefore One-way ANOVA followed by Tukey’s multiple comparison post hoc test was performed.

## Supplementary Material

dvag031_Supplemental_Files

## Data Availability

The data that support the findings of this study are openly available. The epigenetics raw dataset is available in the European Nucleotide Archive (ENA) and is freely accessible under the accession number PRJEB83100. The transcriptomics raw dataset is freely available in the GEO repository under the accession number GSE139599. Finally, the metabolomics raw data can be found in the Zenodo repository under the record number 8297064.
