# Supplementary material for: Integrative multi-omic analysis reveals epigenetic and neurotransmitter signaling disruption induced by tributyltin in zebrafish eleutheroembryos
Source: Environ Epigenet. 2026 Sep 24;12(1):dvag031. doi: 10.1093/eep/dvag031 (PMC13670956; doi:10.1093/eep/dvag031)
Supplement: Supplementary file 1 [file dvag031_supplemental_files.zip › FigureS1.pdf]

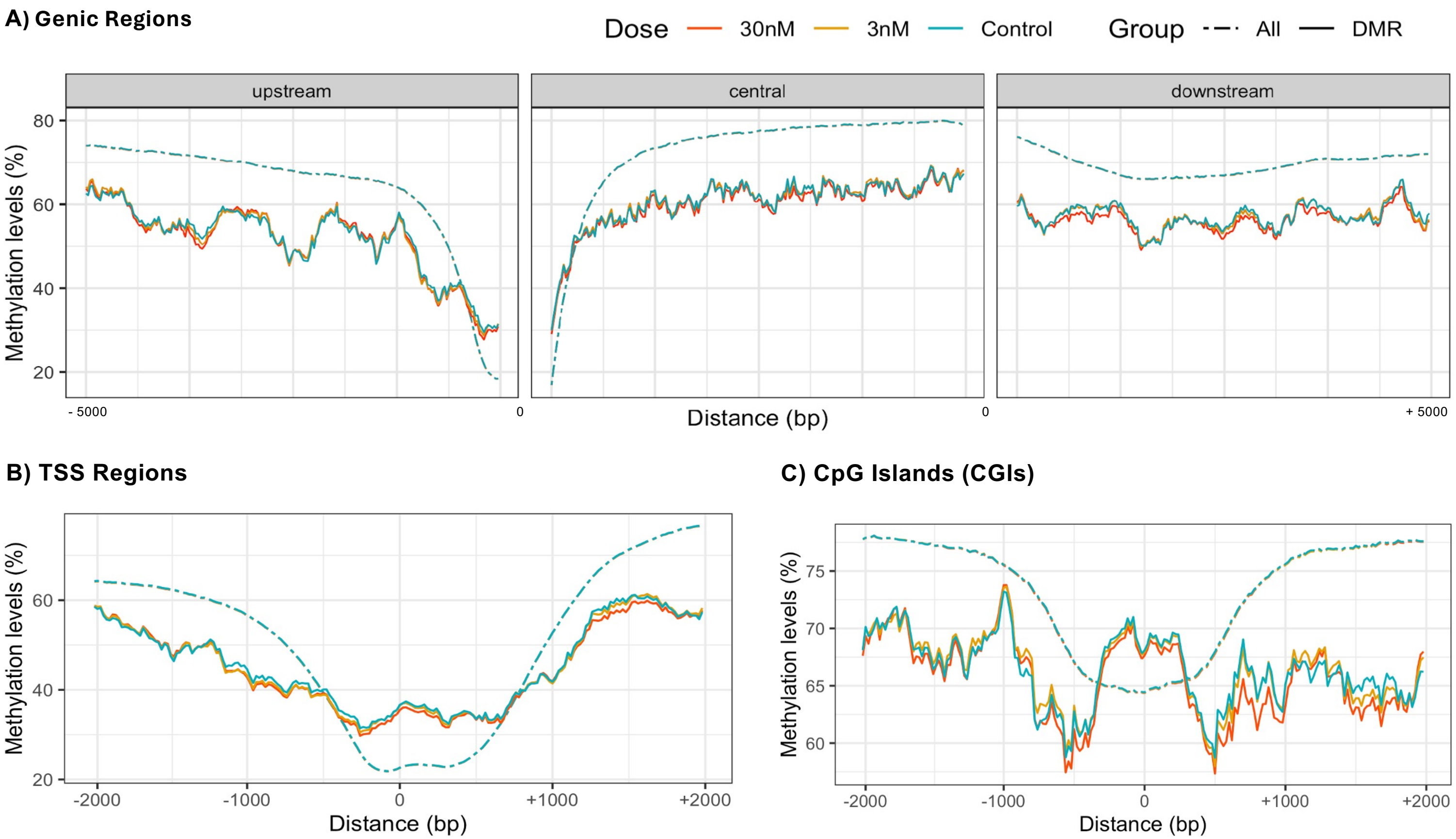
A) Genic Regions
Dose
30nM
3nM
Control
Group
All
DMR
upstream
central
downstream
Methylation levels (%)
80
60
40
20
- 5000
0
Distance (bp)
0
+ 5000
B) TSS Regions
Methylation levels (%)
60
40
20
-2000
-1000
0
+1000
+2000
Distance (bp)
C) CpG Islands (CGIs)
Methylation levels (%)
75
70
65
60
-2000
-1000
0
+1000
+2000
Distance (bp)
