# Supplementary material for: Integrative multi-omic analysis reveals epigenetic and neurotransmitter signaling disruption induced by tributyltin in zebrafish eleutheroembryos
Source: Environ Epigenet. 2026 Sep 24;12(1):dvag031. doi: 10.1093/eep/dvag031 (PMC13670956; doi:10.1093/eep/dvag031)
Supplement: Supplementary file 1 [file dvag031_supplemental_files.zip › FigureS2bis.pdf]

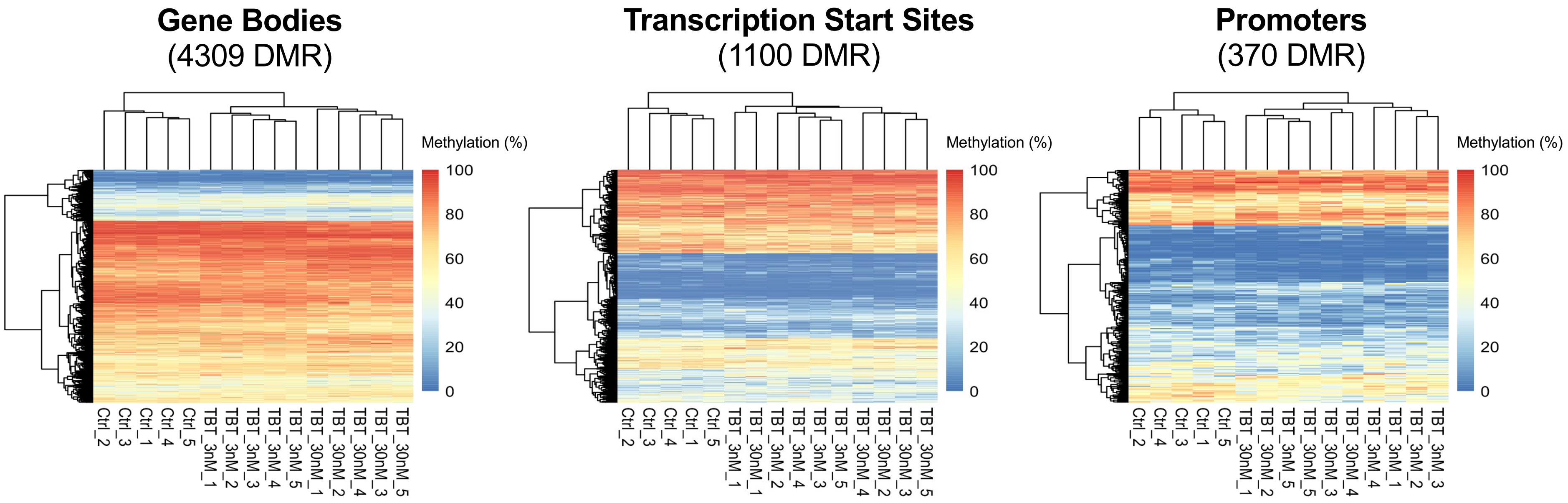
Gene Bodies
(4309 DMR)
Transcription Start Sites
(1100 DMR)
Promoters
(370 DMR)
Methylation (%)
100
80
60
40
20
0
Ctrl_2
Ctrl_3
Ctrl_1
Ctrl_4
Ctrl_5
TBT_3nM_1
TBT_3nM_2
TBT_3nM_3
TBT_3nM_4
TBT_3nM_5
TBT_30nM_1
TBT_30nM_2
TBT_30nM_4
TBT_30nM_3
TBT_30nM_5
